# Accelerated pyro-catalytic hydrogen production enabled by plasmonic local heating of Au on pyroelectric BaTiO_3_ nanoparticles

**DOI:** 10.1038/s41467-022-33818-4

**Published:** 2022-10-17

**Authors:** Huilin You, Siqi Li, Yulong Fan, Xuyun Guo, Zezhou Lin, Ran Ding, Xin Cheng, Hao Zhang, Tsz Woon Benedict Lo, Jianhua Hao, Ye Zhu, Hwa-Yaw Tam, Dangyuan Lei, Chi-Hang Lam, Haitao Huang

**Affiliations:** 1 Department of Applied Physics and Research Institute for Smart Energy, The Hong Kong Polytechnic University, Hong Kong SAR, China; 2 Department of Materials Science and Engineering, The Hong Kong Institute of Clean Energy, The City University of Hong Kong, Hong Kong SAR, China; 3 Information Materials and Intelligent Sensing Laboratory of Anhui Province, Key Laboratory of Opto-Electronic Information Acquisition and Manipulation of Ministry of Education, School of Physics and Materials Science, Anhui University, Hefei, 230601 Anhui China; 4 Department of Electrical Engineering, The Hong Kong Polytechnic University, Hong Kong SAR, China; 5 Department of Applied Biology and Chemical Technology, The Hong Kong Polytechnic University, Hong Kong SAR, China

**Keywords:** Materials for energy and catalysis, Nanoscale materials, Hydrogen energy

## Abstract

The greatest challenge that limits the application of pyro-catalytic materials is the lack of highly frequent thermal cycling due to the enormous heat capacity of ambient environment, resulting in low pyro-catalytic efficiency. Here, we introduce localized plasmonic heat sources to rapidly yet efficiently heat up pyro-catalytic material itself without wasting energy to raise the surrounding temperature, triggering a significantly expedited pyro-catalytic reaction and enabling multiple pyro-catalytic cycling per unit time. In our work, plasmonic metal/pyro-catalyst composite is fabricated by in situ grown gold nanoparticles on three-dimensional structured coral-like BaTiO_3_ nanoparticles, which achieves a high hydrogen production rate of 133.1 ± 4.4 μmol·g^−1^·h^−1^ under pulsed laser irradiation. We also use theoretical analysis to study the effect of plasmonic local heating on pyro-catalysis. The synergy between plasmonic local heating and pyro-catalysis will bring new opportunities in pyro-catalysis for pollutant treatment, clean energy production, and biological applications.

## Abstract (web summary)

While pyrocatalysis offers a means to store energy in chemical bonds from temperature changes, most materials show low conversion efficiencies. Here, authors examine Au nanoparticles on BaTiO3 and demonstrate localized plasmonic heat sources to enhance pyrocatalytic H_2_ evolution.

## Introduction

Pyro-catalysis refers to the catalysis triggered by temperature fluctuation induced pyroelectric surface charges in pyroelectric materials^1,2^, which is a self-powered catalysis technique by harvesting waste energy from the environment. Recently, pyro-catalysis has attracted increasing attention in clean energy production and reactive oxygen species (ROS) generation^3–9^. Xie et al. reported the coupling between pyroelectric effect and electro-chemical process, where pyroelectric polyvinylidene fluoride and lead zirconate titanate (PZT-5H) polycrystalline ceramic thin films were used as a voltage source to electrolyze water^3^. Xiao et al. presented pyro-catalytic CO_2_ reduction by using bismuth tungstate nanoplates, where 55.0 μmol g^−1^ methanol production was achieved after 20 thermal cycles between 15 and 70 °C^5^. The pyroelectric induced surface charges are also capable of generating ROS, such as, hydroxyl (•OH), superoxide (•O_2_^−^), singlet oxygen (^1^O_2_), and hydrogen peroxide (H_2_O_2_)^6–9^. The pyro-catalytic generated ROS can be further used for disinfection and dye treatment. Gutmann et al. reported the impact of thermally excited pyroelectric LiNbO_3_ and LiTaO_3_ nano- and microcrystalline powders for the disinfection of *Escherichia Coli* in aqueous solutions, where a high antimicrobial activity was observed^7^. Wu et al. reported the pyro-catalytic decomposition of Rhodamine B (RhB) solution by making use of the hydrothermally synthesized BiFeO_3_ nanoparticles (NPs)^8^. The RhB dye was almost completely decomposed after undergoing 85 thermal cycles between 27 and 38 °C.

However, the currently available pyroelectric materials, whose pyro-catalytic capability relies on the variation of ambient temperature, show low pyro-catalytic efficiencies. Under steady state, the short-circuit pyro-current (*I*) available for pyro-catalytic reactions can be calculated as^10^:

1 $$I=p\cdot A\cdot {dT}/{dt}$$

where *p* is the pyroelectric coefficient, *A* is the area of the surface that is normal to the polarization direction, and *dT/dt* is the temperature change rate. Assuming a pyroelectric coefficient of 10–50 nC cm^−2^ K^−1^ and a typical low environmental temperature ramping rate of 0.1 K s^−1^ ^11–14^, the maximum pyroelectric current for pyro-catalytic reaction is only around 1–5 nA cm^−2^, which is about 2–3 orders of magnitude lower than that of photocatalysts^15^. Simply increasing the temperature ramping rate *dT/dt* does not help since the total available charge for catalysis during one temperature ramping or cooling is *Q* = *p·A·*Δ*T*, (direct time integral of Eq. 1), showing that the pyro-catalytic reaction depends on the temperature change Δ*T* per thermal cycle. Considering that the environmental temperature change is always quite limited, the only way to increase the pyro-catalytic production rate is to increase the number of temperature cycling. However, due to the huge heat capacity of the surrounding media, it is still a great challenge to achieve multiple thermal cycling of the pyro-catalyst within a short time interval using macroscopic heating^16^.

To create multiple temperature cycling at the least expense of input thermal energy, it would be ideal to have a localized heat source that only heats up the pyro-catalytic material itself to a certain degree while maintains the surrounding temperature almost unchanged. Plasmonic nanostructures that absorb light and convert it into heat are one of such ideal candidates. The localized heat generated by thermo-plasmonic nanostructures can be easily fine-tuned, turned on or off by external light irradiation, which act as rapid, dynamic, and controllable localized heat sources^17,18^. It has been reported that under the illumination of a 532 nm laser (excitation power of 5 W), there will be a temperature change of around 100 K within a distance of around 1 µm of a Au nanoparticle (around 40 nm) in a timescale of 10–100 µs^19^. Such a large temperature rise in an ultrashort timescale would provide an ideal environment for pyro-catalysis.

Herein, we select barium titanate (BaTiO_3_) as the model material to investigate the highly efficient and greatly accelerated pyro-catalysis enabled by plasmonic local heating. Among all ferroelectric materials, BaTiO_3_ exhibits a large pyroelectric coefficient (*p*) of about 20–30 nC cm^−2^ K^−1^ and has been widely investigated as the lead-free perovskite materials for pyroelectric applications^20–22^. As compared with the thick film or bulk counterparts, the use of NPs can greatly enhance the specific surface area of the pyroelectric material and hence increase the available pyroelectric charges^23^. In this work, BaTiO_3_ NPs were decorated with Au NPs as the plasmonic heat sources, which possess appealing characteristics such as simple structure, easily tunable morphology, and superior photo-thermal conversion efficiency. As one of the most attractive catalytic reactions, hydrogen production from water splitting was used to validate our hypothesis of plasmonic local heating accelerated pyro-catalysis.

In this work, three-dimensional hierarchically structured coral-like BaTiO_3_ NPs were first synthesized via a hydrothermal method and then coated with in situ grown Au NPs (named as Au/BaTiO_3_ hereafter). The plasmonic/semiconductor nano reactors have demonstrated an accelerated pyro-catalytic hydrogen production rate of around 133.1 ± 4.4 μmol g^−1^ h^−1^ by the thermos-plasmonic local heating under irradiation of a nanosecond laser at the wavelength of plasmonic resonance of Au NPs (532 nm). The extremely rapid heating and cooling enabled by the plasmonic local heating and subsequent environment cooling will bring new opportunities for the applications of efficient pyro-catalysis in biological treatment, clean energy production and pollutant removal.

## Results

### Characterization of material

**Fig. 1:**
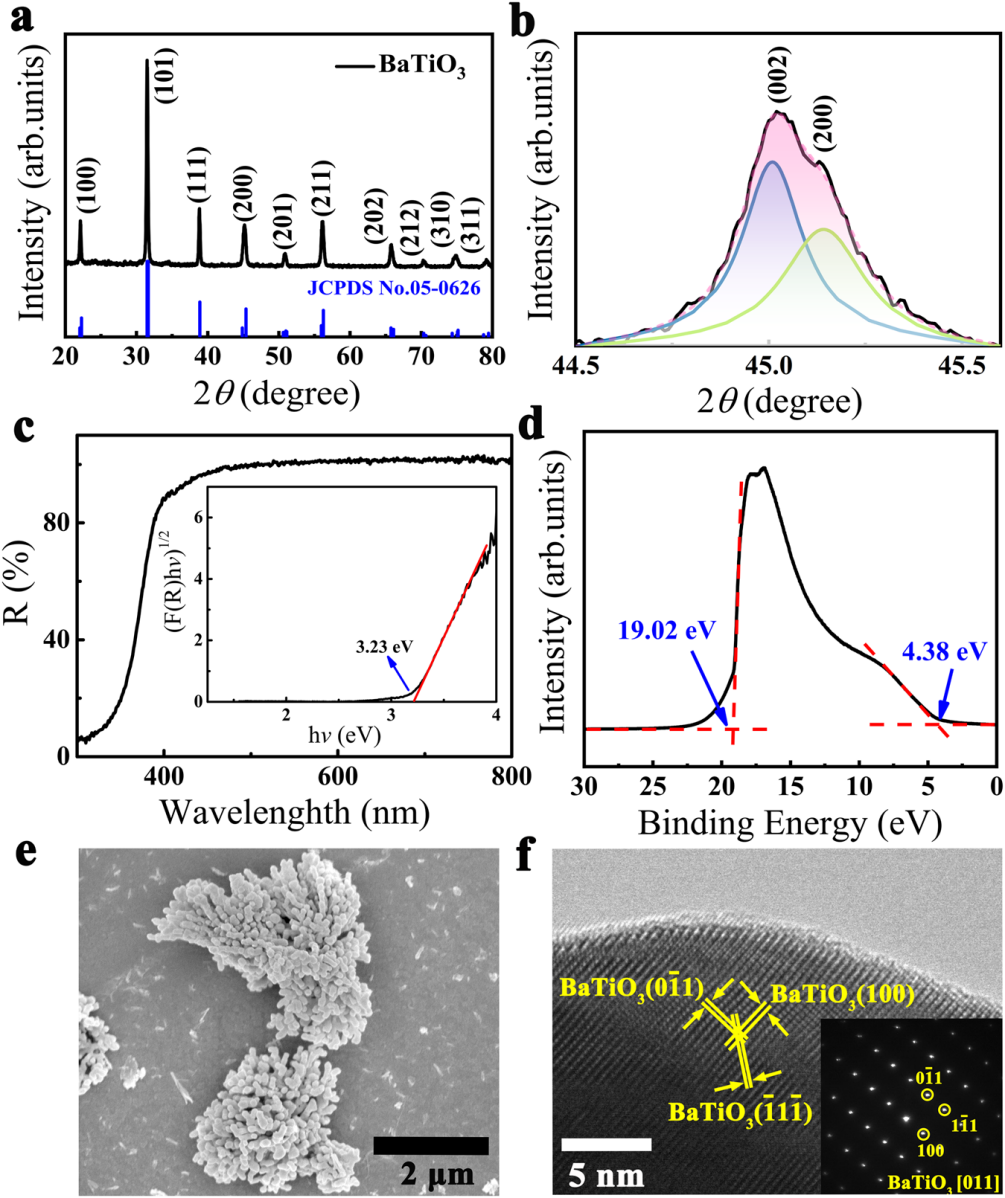
Characterizations of as-prepared BaTiO_3_ NPs. **a** XRD spectra. **b** Enlarged (002) and (200) XRD peaks. **c** UV-Vis diffuse reflectance spectra with Tauc’s plot as inset. **d** UPS. **e** SEM image. **f** HRTEM image. The inset in (**f**) is the SAED pattern along [011] zone axis. Source data are provided as a Source Data file.

As shown in Fig. 1a, all the diffraction peaks observed in X-ray diffraction (XRD) patterns can be assigned to a pure perovskite phase with a point group 4 *mm* (JCPDS Card No. 05-0626). The diffraction peaks suggest a tetragonal phase of the as-synthesized BaTiO_3_ sample, implying that it is ferroelectric^24,25^. The tetragonality (*c/a* ratio of the lattice parameters) of the BaTiO_3_ NPs can be estimated from the split of the {200} peaks in the XRD pattern (Fig. 1b), which is around 1.003 (Supplementary Table 1). This value is smaller than the bulk counterpart due to the size effect^26^ that usually suppresses the ferroelectricity at the nanoscale range. The ferroelectricity of the synthesized BaTiO_3_ sample is further verified by piezoresponse force microscope (PFM) in a dual AC resonance tracking mode. As shown in Supplementary Fig. 1, the butterfly-like hysteresis loops and phase switching of 180° demonstrate the ferroelectricity of the BaTiO_3_. To gain a complete understanding of the band structure, ultraviolet-visible (UV-Vis) diffuse reflectance spectra and ultraviolet photoelectron spectroscopy (UPS) spectra were conducted, as shown in Fig. 1c and d. According to Supplementary Eqs. 3 and 4, the band gap of our synthesized BaTiO_3_ is 3.23 eV (inset of Fig. 1c), corresponding to a photon energy of ultraviolet light. In Fig. 1d, the low binding energy region (E_low-binding_) and the secondary electron cut-off energy (E_cutoff_) observed from the UPS spectra of BaTiO_3_ are 4.38 and 19.02 eV, respectively. Through UPS measurement, the valance band maximum (VBM) can be estimated by, VBM = *hν*−(E_cut-off_ − E_low-binding_), where *hν* is photon energy (21.22 eV). Thus, the valence band edge and conduction band edge of BaTiO_3_ sample are 2.14 and −1.09 eV, respectively, suggesting that the synthesized BaTiO_3_ material has a suitable energy band structure for water splitting. The scanning electron microscopy (SEM) image (Fig. 1e) shows that the as-prepared BaTiO_3_ NPs possess a morphology of three-dimensional hierarchically structured coral-like shape with an average size of several hundred nanometers. The high-resolution transmission electron microscopy (HRTEM) image (Fig. 1f) and high-angle annular dark field-scanning TEM (HAADF-STEM) image (Supplementary Fig. 2) of the NPs show three characteristic lattice fringes, which agree well with the *d*-spacings of the (100), (0$\bar{1}$1) and ($\bar{1}$1$\bar{1}$) planes of BaTiO_3_. Its selected area electron diffraction (SAED) pattern (inset of Fig. 1f) is clear and sharp, manifesting high crystallinity of the synthesized BaTiO_3_ NPs that agrees well with the XRD pattern (Fig. 1a). The specific surface area of the synthesized BaTiO_3_ NPs is around 38.85 m^2^/g, according to Brunauer-Emmett-Teller (BET) surface area measurement (Supplementary Fig. 3).

**Fig. 2:**
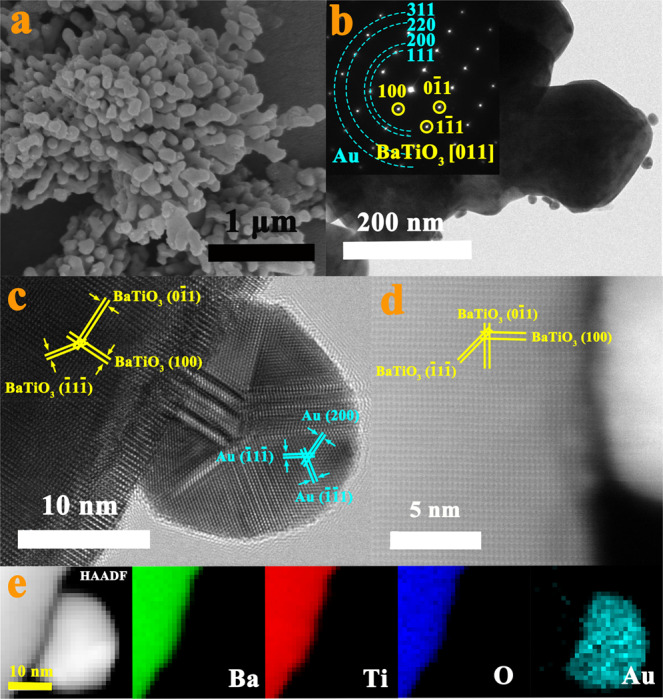
Morphology characterizations of Au/BaTiO_3_ NPs. **a** SEM image. **b** TEM image with SAED pattern as inset. **c** HRTEM image. **d** HAADF-STEM image with atomic structure as inset. **e** HAADF-STEM image and corresponding elemental mapping of Ba, Ti, O, and Au elements.

The SEM and TEM image of Au/BaTiO_3_ hybrid nanostructures are shown in Fig. 2a and b, respectively. The in situ synthesized Au NPs possess a nanosphere morphology with an average size of around 18 nm (Supplementary Fig. 4). The SAED pattern (inset of Fig. 2b) along the zone axis of [011] also verifies the successful synthesis of Au/BaTiO_3_ hybrid nanostructures. The HRTEM image (Fig. 2c) of the nanoparticle shows the characteristic lattice fringes that agree well with the *d*-spacings of the (100), (0$\bar{1}$1) and ($\bar{1}$1$\bar{1}$) planes of BaTiO_3_ and those of (200), $(\bar{1}\bar{1}1)$ and ($\bar{1}$1$\bar{1}$) planes of Au. The HAADF-STEM image of the BaTiO_3_ NPs after the growth of Au NPs is presented in Fig. 2d, where the three characteristic *d*-spacings of the (100), (0$\bar{1}$1) and ($\bar{1}$1$\bar{1}$) planes of BaTiO_3_ can be clearly seen. This reveals that the growth of Au NPs on the surface of BaTiO_3_ does not destroy the structure of BaTiO_3_. The HAADF-STEM image and the associated elemental mappings of Ba, Ti, O and Au (Fig. 2e) show uniform distribution of these elements. The successful synthesis of Au decorated BaTiO_3_ NPs could also be verified via UV-visible spectra in Supplementary Fig. 5, where the characteristic absorption peak of Au NPs was found to locate at around 532 nm.

### Pyro-catalytic hydrogen evolution from water splitting

**Fig. 3:**
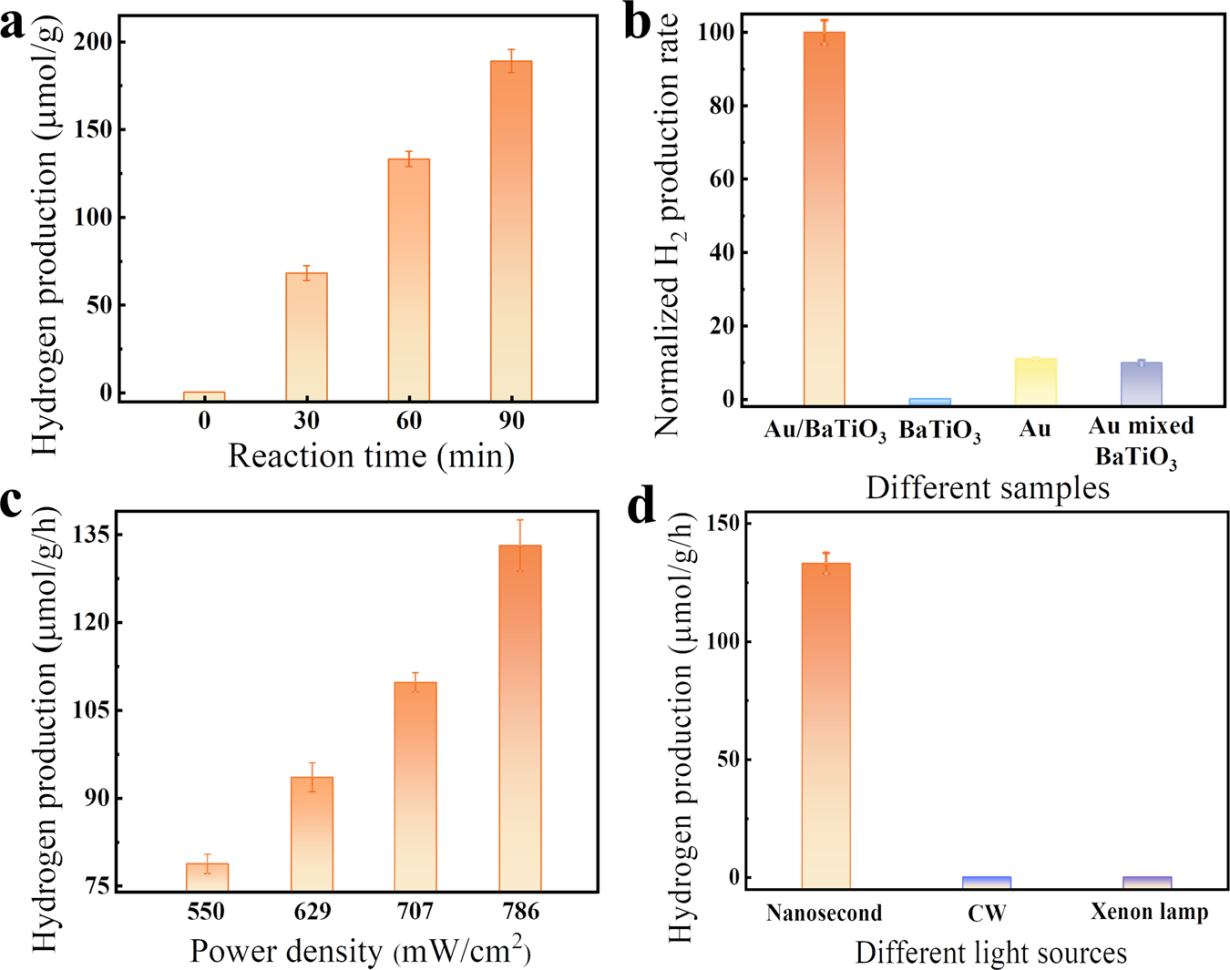
H_2_ generation from water splitting by Au/BaTiO_3_ NPs. **a** Hydrogen generation under different reaction time. **b** Normalized H_2_ production rate (normalized to the production rate of Au/BaTiO_3_ NPs) of different samples under the irradiation by a 532 nm nanosecond laser. **c** Hydrogen generation under the irradiation of a 532 nm nanosecond pulsed laser with different power densities. **d** H_2_ generation by Au/BaTiO_3_ NPs illuminated under different light sources. The error bars represent the standard deviation of three parallel experiments. Source data are provided as a Source Data file.

Figure 3a reveals the thermo-plasmon induced pyro-catalytic hydrogen generation by Au/BaTiO_3_ NPs, where the total hydrogen production under the irradiation power of 786 mW cm^−2^ for 60 min is up to 133.1 ± 4.4 μmol g^−1^. The H_2_ and O_2_ generated from full water splitting by Au/BaTiO_3_ NPs under the irradiation of a 532 nm nanosecond laser is shown in Supplementary Fig. 6 and the results are compared with other reported data (Supplementary Table 2). The morphology of the BaTiO_3_ NPs after 90 min pyro-catalysis (Supplementary Figs. 7 and 8) shows almost no change, indicating that the Au/BaTiO_3_ NPs have good stability toward pyro-catalytic hydrogen production.

The control experiment in Fig. 3b shows that the BaTiO_3_ NPs do not present any noticeable hydrogen production via photocatalysis due to its large band gap of 3.23 eV (Fig. 1c). It can be seen from Fig. 3b that Au/BaTiO_3_ NPs exhibit much higher hydrogen production rate than that of Au NPs alone, which indicates that the plasmonic induced catalytic water splitting (such as the reported water dissociation by plasmonic induced hot carriers)^27,28^ makes a minor contribution in the present case. From Fig. 3b, it can also be found that, for Au NPs physically mixed with BaTiO_3_ NPs, the catalytic water splitting is slightly less than pure Au NPs, indicating that, in this physical mixture of NPs, the catalytic water splitting mainly comes from Au NPs and the light scattering of BaTiO_3_ NPs may even slightly degrade the catalytic performance. This result emphasizes the importance of good thermal contact between Au NPs and BaTiO_3_ NPs in order to realize the plasmonic local heating accelerated pyro-catalytic water splitting. It can also be concluded that, for Au/BaTiO_3_ NPs, the pyro-catalytic H_2_ production makes the most significant contribution to the overall hydrogen production, rather than photocatalysis and hot electron driven water splitting.

Since the total available charge for pyro-catalysis during one temperature ramping or cooling is *Q* = *p·A·*Δ*T*, to have a large hydrogen production, not only the temperature change (Δ*T*) should be high, but also the number of thermal cycling should be enough. The nanosecond pulsed laser has both high peak power density to generate a sufficiently large Δ*T* through plasmonic local heating (which will be discussed below), and enough thermal cyclings per unit time (10 cycles per second in the current work, corresponding to a laser pulse repetition rate of 10 Hz). With increasing average power density of the laser pulse, Δ*T* will be increased through the plasmonic photo-thermal conversion^29^, leading to higher hydrogen production (Fig. 3c).

To reveal the importance of thermal cycling, we compare the hydrogen production by illuminating the Au/BaTiO_3_ NPs with different light sources. Though the average power density of a continuous wave (CW) laser (14.15 W cm^−2^) and a xenon lamp (694 mW cm^−2^) are much higher or very close to that of a nanosecond laser (786 mW cm^−2^), no detectable hydrogen production under the illumination of these two light sources can be found (Fig. 3d). This can be ascribed to the fact that the temperature change of the plasmonic NPs induced by CW laser or xenon lamp irradiation is too low. For Au NPs with a radius of 10 nm to have 5 °C increase in temperature, a power density of 1 × 10^5^ W cm^−2^ is needed^30^. Besides, the raised temperature will be kept almost constant under the continuous illumination of a CW laser or a xenon lamp. This is in strong contrast to the case of a pulsed laser. For a nanosecond laser, due to the fast energy releases in several nanoseconds, a very high peak power density up to 5.09 × 10^6^ W cm^−2^ (average power density of 786 mW cm^−2^) can be achieved within 24 ns, resulting in a large temperature rise on the Au/BaTiO_3_ NPs within a short time. Moreover, the time interval between successive laser pulses of the nanosecond laser is much longer than that of the laser pulse itself, which allows enough time for the heated BaTiO_3_ NPs to cool down by the surrounding liquid, and make them ready for the next thermal cycle stimulated by the next laser pulse. It can be predicted that, by increasing the laser pulse repetition rate, the hydrogen production rate can be further increased due to increased number of thermal cycling per unit time.

### Thermal simulation

To better understand the mechanism of plasmon induced localized pyro-catalysis, commercial full-wave finite element method simulations by COMSOL RF and Multiphysics 5.5 modules were performed. The simplified structural model is depicted in Supplementary Fig. 9, where the temperature distribution profile of the Au nanosphere (9 nm in radius) grown on BaTiO_3_ NP can be obtained.

**Fig. 4:**
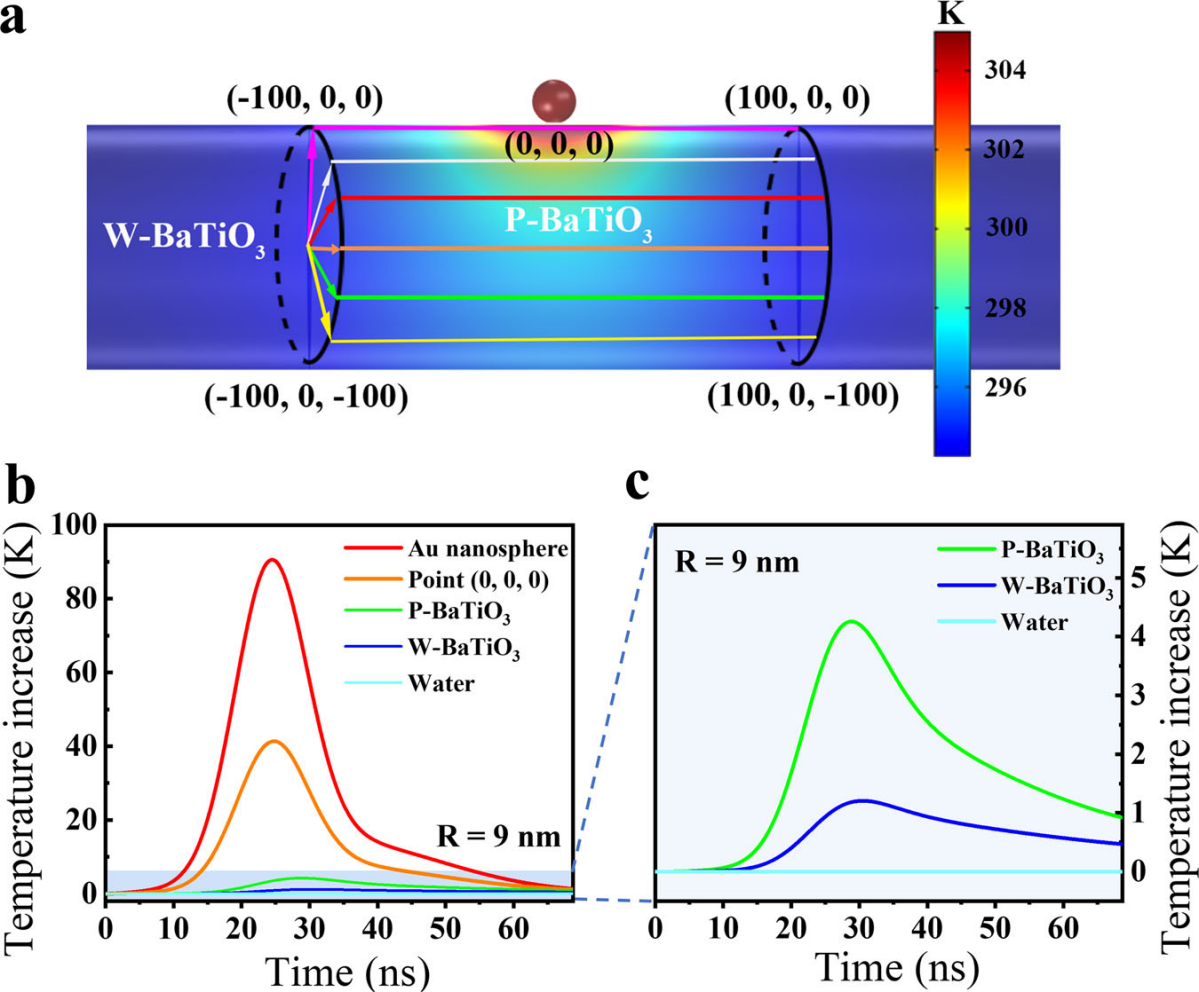
Thermal simulation. **a** Temperature distribution of the structural model of a Au NP (9 nm in radius) suspending over a BaTiO_3_ cylinder with length of 1 µm and radius of 50 nm at the moment with highest temperature inside Au NP. **b** Time evolution of the temperature of point (0, 0, 0) and the average temperature of Au NP, region of P-BaTiO_3_, region of W-BaTiO_3_, and surrounding water. **c** Enlarged time evolution of the average temperatures of the regions of P-BaTiO_3_, W-BaTiO_3_, and surrounding water. Source data are provided as a Source Data file.

The heat absorbed by BaTiO_3_ NP or surrounding water largely comes from calefactive Au NP through heat conduction. Note that although thermal convection does exist in any stirred system, the globally unchanged temperature (will be demonstrated in the following part) of the surrounding water allows us to neglect this heat transfer process to simplify our quantitative study on the effect of local heating induced pyroelectricity. A structural model for the calculation of temperature distribution of Au decorated BaTiO_3_ NP is shown in Fig. 4a, where an average value of 9 nm is typically taken for the radius of Au NP, according to the experimental value (Supplementary Fig. 4). The point (0, 0, 0) is defined as the surface position of BaTiO_3_ just beneath the center of Au nanosphere. P-BaTiO_3_ denotes the region of BaTiO_3_ NP (the cylindrical region with a length of 200 nm and a radius of 50 nm), and W-BaTiO_3_ is the whole BaTiO_3_ NP (a cylinder with a length of 1 µm and a radius of 50 nm). Surrounding water denotes the water sphere which surrounds the whole Au/BaTiO_3_ with diameter of 20 μm. Figure 4b and c show the time evolution of the temperatures at different regions of a Au/BaTiO_3_ NP and the surrounding water. The effect of the radius of Au nanosphere on the temperature variation can be seen in Supplementary Fig. 10, which agrees with other groups’ observations^30^. It can be concluded from Fig. 4 and Supplementary Fig. 10 that the temperature changes of Au nanosphere and P-BaTiO_3_ increase with increasing radius of decorated Au nanospheres, while those of W-BaTiO_3_ and surrounding water show no obvious increase. This result is verified by direct monitoring of the water temperature during 90 min of nanosecond laser irradiation (Supplementary Fig. 11) with a thermometer. On the BaTiO_3_ NP, the temperature rise at a position closer to the Au nanosphere is also larger (Supplementary Fig. 10e).

Moreover, when the lase pulse is off, the Au and BaTiO_3_ NPs cool down to room temperature in a period of about 50 ns (Fig. 4b) due to the small total heat capacity of the localized plasmon heated region as compared with that of the vast surroundings. It should be emphasized that no matter heating or cooling, pyro-catalytic reaction will take place so long as the temperature is changed. Therefore, the plasmonic localized heating enables multiple thermal cycles within a short time period due to its rapid heating and cooling processes, which is advantageous to the efficient increase of the overall pyro-catalytic reaction product and reaction rate.

## Discussion

**Fig. 5:**
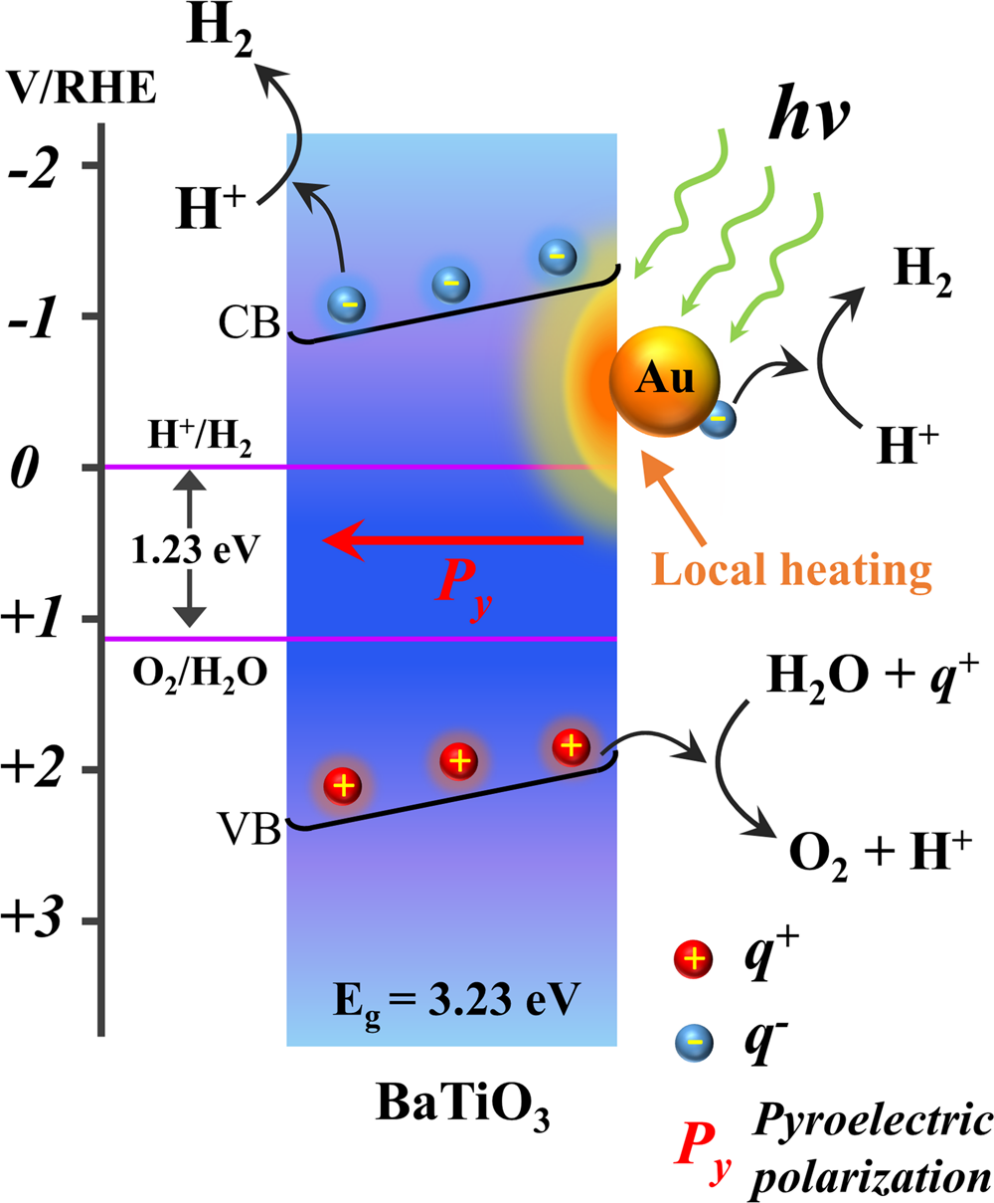
Pyro-catalytic mechanism. Schematic illustration of pyro-catalytic hydrogen generation of Au/BaTiO_3_ NP driven by surface plasmon local heating.

A schematic illustration of pyro-catalytic hydrogen generation of Au/BaTiO_3_ NP driven by surface plasmon induced local heating is shown in Fig. 5. Under illumination, the surface plasmon resonance of a Au NP induces a rapid increase in the local temperature of its attached BaTiO_3_ NP. The uncompensated pyroelectric charges on the BaTiO_3_ surface can react with surrounding water molecules to generate hydrogen and oxygen, which is discussed in detail in Supporting Information (Supplementary Fig. 12). When the illumination is off, the BaTiO_3_ will undergo a cooling cycle with rapid dissipation of the generated heat into the surrounding water. Again, the uncompensated pyroelectric surface charges during the cooling cycle will participate in the pyro-catalytic water splitting. Moreover, the internal electric field built up from the surface pyroelectric charges can further facilitate the charge separation and charge transfer for pyro-catalytic hydrogen and oxygen production.

In addition to the pyro-catalysis of BaTiO_3_ and hot electron catalysis of Au, thermoelectric effect could also contribute to the overall catalytic reaction of water splitting. Thus, we perform thermal calculation by COMSOL Multiphysics 5.5 to estimate the thermoelectric effect of Au/BaTiO_3_. A simplified structural model (Supplementary Fig. 13) is used to calculate the quantity of charges released due to the thermoelectric effect (Supplementary Fig. 14).

As a comparison, for pyroelectric materials, the quantity of electrons induced by pyroelectric effect can be expressed by as^31^,

2 $$Q=p\cdot \int \triangle T\left(\hat{{{{{{\bf{n}}}}}}}\,\cdot\, \hat{{{{{{\bf{P}}}}}}}\right){dA}$$

where *p* is the pyroelectric coefficient with a value between 20 and 30 nC cm^−2^ K^−1^, Δ*T* is the temperature rise in heating (or drop during cooling), $\hat{{{{{{\bf{n}}}}}}}$ is the unit vector along the surface normal direction, and $\hat{{{{{{\bf{P}}}}}}}$ is the unit vector along spontaneous polarization direction. The surface integral of Δ*T*$(\hat{{{{{{\bf{n}}}}}}}\cdot \hat{{{{{{\bf{P}}}}}}})$*dA* with a corresponding spontaneous polarization perpendicular to the axial direction is shown in Supplementary Fig. 15. Taking the pyroelectric coefficient (*p*) of 30 nC cm^−2^ K^−1^ as an example, we can easily calculate that the pyroelectric charges in one heating/cooling cycle are 5.12 × 10^−17^ C, which are much larger than those due to thermoelectric effect (Supplementary Fig. 14). Therefore, the thermoelectric effect due to the non-uniform local heating by the plasmonic NPs can be neglected. Here, the photocatalytic contribution to the water splitting can also be excluded since BaTiO_3_ has a large band gap located in the ultraviolet range.

To evaluate the pyro-catalytic hydrogen generation performance of BaTiO_3_, we calculate the the total available pyroelectric charges using Eq. 2 (see supporting information for detailed calculation). In general, to increase the pyro-catalytic H_2_ generation rate, we can either reduce the size of BaTiO_3_ NP or replace the current laser source by one with a higher repetition rate. Our above discussion is based on the assumption that there is only one Au NP on a single BaTiO_3_ particle. If there are multiple optically isolated Au NPs decorated on a single BaTiO_3_ particle, the heating effect will be algebraically augmented and the pyro-catalytic H_2_ production rate will be further increased. If some of the Au NPs are closely packed to trigger electromagnetic coupling, then the heating effect will be more than simple algebraic summation. The above estimation does not take into consideration the possible factors that may restrict the maximum achievable pyro-catalytic H_2_ production rate, such as surface charge loss by capacitive effects, insufficient absorption of the incident light, and charge recombination, etc^32,33^. We also want to emphasize that the pyro-catalytic hydrogen production reported here cannot be directly benchmarked to the state-of-the-art photocatalytic hydrogen production from water splitting since the underlying mechanisms are different.

Apart from hydrogen production, hydroxyl radicals are also detected during the pyro-catalysis (Supplementary Fig. 16), which can be used for biological applications such as cancer therapy^34–37^. Localized pyro-catalysis is effective only in nanoscale range and does not affect the surroundings, which is an attractive feature for achieving accurate treatment in tumor cell, leading to minor side effects. The wavelength of the excitation light can also be adjusted to near infrared band (biological window) through changing the morphology of Au NPs, for instance, utilizing gold nanorods. Decreasing the diameter of laser beam to µm can largely decease the power needed for plasmon induced pyro-catalysis.

In summary, we have demonstrated the greatly accelerated pyro-catalytic hydrogen production by coupling pyroelectric material with thermal-plasmonic one: three-dimensional hierarchically structured coral-like BaTiO_3_ NPs were capped by in situ grown Au NPs. A high hydrogen production rate of 133.1 ± 4.4 μmol g^−1^h^−1^ was achieved under the irradiation of a 532 nm nanosecond laser with 0.5 W optical power. The pulsed laser irradiation brings about a dramatically rapid local heating under pulsed excitation, and a fast cooling during pulse off period, thus greatly promoting and accelerating the overall pyro-catalytic hydrogen production. The synergy between plasmonic local heating effect and pyro-catalysis will open up new avenues for efficient catalysis for biological applications, clean energy production and pollutant treatment, etc.

## Methods

### Synthesis of BaTiO_3_ and Au decorated on BaTiO_3_ nanoparticles (NPs)

BaTiO_3_ NPs were synthesized through a hydrothermal method^38^. Typically, 1.25 g titanium dioxide (TiO_2_) (Sinopharm Chemical Reagent Co. Ltd.) and 24.08 g sodium hydroxide (NaOH) (International Laboratory USA) were dissolved into 60 mL of deionized water. After stirring for 30 min, the mixed solution was transferred into a 100 mL Teflon-lined stainless-steel autoclave, heated at 180 °C for 24 h, and then cooled down to room temperature naturally to get sodium titanate (Na_2_Ti_3_O_7_). The obtained material was then dispersed into HCl solution (pH = 1) for 4 h to obtain hydrogen titanate (H_2_Ti_3_O_7_). After repeated wash with deionized (DI) water, H_2_Ti_3_O_7_ was washed to pH = 7 and dried at 60 °C for 12 h. After that, 0.1288 g H_2_Ti_3_O_7_ and 0.95 g barium hydroxide (Ba(OH)_2_·8H_2_O) (Sinopharm Chemical Reagent Co. Ltd.) were dissolved in 60 mL of deionized water. The mixed solution was set in a 100-mL Teflon-lined stainless-steel autoclave at 210 °C for 85 min and cooled down to room temperature naturally. Then, barium titanate (BaTiO_3_) was collected by centrifugation and washed with DI water and ethanol several times until pH = 7. Finally, BaTiO_3_ catalyst was obtained after dying at 60 °C for 12 h. The Au/BaTiO_3_ NPs were synthesized in situ via a citrate reduction method. In the synthesis of the Au/BaTiO_3_ NPs, 16.5 mg BaTiO_3_ NPs, 750 µL sodium citrate (40 mmol L^−1^) (International Laboratory USA) and 500 µL HAuCl_4_ (10 mmol L^−1^) (Sigma-Aldrich) were added into 19 mL water. After 30 s ultrasonication, the solution was heated to reach boiling point for several minutes (around 10–12 min) under vigorous stirring to obtain the Au/BaTiO_3_ NPs (until the color the solution turns to be purplish red). For hydrogen production experiment, 3 mL Au/BaTiO_3_ NPs solution were collected by centrifugation at 6000 *rpm* and dispersed in 12 mL water. After 10 s ultrasonication, the obtained catalyst was added into the reactor for H_2_ production. All reagents used as starting materials were of analytical grades.

### Characterization of catalyst

The XRD patterns of synthesized BaTiO_3_ were recorded by Rigaku SmartLab 9KW X-ray powder diffractometer (scan rate of 0.1°/min, scan range of 20–80 degree and the wavelength of the XRD radiation of *λ*~1.54 Å). The morphology of BaTiO_3_ was studied by a TESCAN MAIA3 SEM, where the catalyst was dispersed on carbon conducting paste. Transmission electron microscopy (TEM) and scanning TEM (STEM) were performed using JEOL JEM-2100F TEM/STEM operated at 200 kV, where the catalyst was dispersed in ethanol and then transfer into cupper grid. Electron energy-loss spectroscopy (EELS, by Gatan Enfina) mapping was carried out under 200 kV accelerating voltage with a 13 mrad convergence angle for the optimal probe condition. Energy dispersion of 0.5 eV per channel and 21 mrad collection angle were set up for EELS. High-angle annular dark field (HAADF) images were acquired with an 89 mrad inner angle simultaneously. For High-resolution scanning transmission electron microscopy (HRSTEM), the HAADF detector collection inner angle was set to 41 mrad to increase the S/N ratio. Piezoresponse force microscopy (PFM, Asylum MFP 3D Infinity) was used to characterize the ferroelectricity of the BaTiO_3_ catalyst, where the catalyst was dispersed on Pt coated silicon sheet. UV-Vis Diffuse reflectance spectra was tested by Shimadzu UV-2550 UV-vis spectrophotometer. The UPS was measured by thermo Fisher Nexsa X-ray photoemission spectroscopy, where the catalyst was dispersed on Pt coated silicon sheet (photon energy of 21.22 eV and bias voltage of −10 V). Brunauer-Emmett-Teller (BET) specific surface area analysis was conducted via surface area and porosity analyzer (Micromeritics, ASAP 2020). Power density of one laser pulse was measured via a digital storage oscilloscope (Keysight, Infiniium S-Series, 1 GHz, 20 GSa·s^−1^, 10-bit ADC) with biased silicon detector (EOT, ET-2030, Bandwidth >1.2 GHz, Risetime<300 ps). Hydroxyl was detected via photoluminescence (PL) spectra via Edinburgh FLS920 spectrofluorometer with 321 nm UV light.

### Hydrogen production experiments

The pyro-catalytic hydrogen production of the Au decorated BaTiO_3_ NPs was evaluated offline. In pyro-catalytic experiment, around 2.62 mg Au decorated BaTiO_3_ NPs were dispersed in 12 mL deionized water. The aqueous suspension was put into a 30 mL pear-shaped quartz reactor and sealed using septa in advance, which was then evacuated and purged by N_2_ for about 20 min to completely remove air. A 500 mW 532 nm nanosecond pulsed laser (Continuum, Inlite II) with repetition rate of 10 HZ and pulse width of around 12.7 ns (see Supplementary Fig. 9c for detailed information), a 1 W 532 nm continuous laser (Honkoktech, PSU-H-LED) and a 300 W high-pressure Xenon lamp (Perfect Light, PLS-SXE300) were utilized as light sources. The distance between irradiation source and reactor is 10 cm. To detect the amount of hydrogen production, 300 µL gas component within the reactor was intermittently extracted and injected into a gas chromatograph (Agilent 7890B) with a thermal conductivity detector (chromatographic column: Agilent, length of 30 m and diameter 0.32 mm). 300 µL N_2_ gas is refilled into reactor after sampling, which was taken into consideration in the calculation. The amount of hydrogen gas produced was calculated using a calibration curve (*y* = 137.78*x* + 18.95, *R*^2^ = 0.99962) of hydrogen concentration versus peak area (working range: 300–15,000 ppm; limit of detection: 50 ppm; limit of quantification: 150 ppm). All the hydrogen production experiments were performed three times per experimental parameter set.

### Simulation details

The optical modelling of the plasmonic local heating effect was carried out by two steps of simulation by using the commercial full-wave Finite Element Method software (COMSOL RF and Multiphysics 5.5). A circularly polarized plane wave was used as the background field to interact with the faceted gold nanospheres with different radii, i.e., 6, 9, and 12 nm suspended over a BaTiO_3_ cylinder. BaTiO_3_ nanowire is placed 1 nm below the faceted gold nanospheres. The absorption cross section was thus obtained as the absorbed energy normalized to the incident optical intensity. PML condition was applied to the outer surface of water domain. The plasmonic local heating effect was studied by using the COMSOL Multiphysics 5.5, Heat Transfer module. The geometries and the relative position of the faceted gold nanospheres and BaTiO_3_ cylinder are all set the same as the optical modelling except that they are surrounded by a water sphere with 20 μm in diameter, which is large enough to render the average temperature rise negligible during the one-pulse excitation, complying with the experimental observation. The gold nanospheres are modeled as heat sources whose heating power equals to the time dependent laser power density times their absorption cross sections obtained from the optical modelling. The heat transfer coefficient 1000 W m^−2^ K^−1^ of the convective heat flux was assigned to the outer surface of the water sphere, a commonly used value describing the stirring process. The mesh size in the nanosphere and that in the Part BaTiO_3_ (P-BaTiO_3_, the cylindrical region with a length of 200 nm and a radius of 50 nm) are set to be a constant (2 nm). The mesh size in the whole BaTiO_3_ (W- BaTiO_3_, a cylinder with a length of 1 µm and a radius of 50 nm) and that in the water sphere gradually increase from 2 nm to 20 nm and from 2 nm to 500 nm respectively, to accelerate the simulation. In this study, a point probe at (0, 0, 0) has been used to measure the temperature rise of the point on the BaTiO_3_ surface but right underneath the gold nanosphere, and four domain probes has been used to monitor the average temperature rises in the gold nanosphere, P-BaTiO_3_, W-BaTiO_3_ and the water sphere, respectively. The domain probe can integrate the temperature rise within the probed domain and divide it by the volume, thus giving us directly the average temperature rises during the whole heating process. Time step was set to 0.1 ns in order not to lose any information. The position of the polarization axis within the particle was in axial direction.

## Supplementary information

Supplementary Information

Peer Review File

## Data Availability

Source data are provided with this paper.

## Source data

Source Data

## References

[1] Zhang Y (2020). Thermal energy harvesting using pyroelectric-electrochemical coupling in ferroelectric materials. Joule.

[2] You H (2018). Room-temperature pyro-catalytic hydrogen generation of 2D few-layer black phosphorene under cold-hot alternation. Nat. Commun..

[3] Xie M, Dunn S, Boulbar EL, Bowen CR (2017). Pyroelectric energy harvesting for water splitting. Int. J. Hydrog. Energy.

[4] Zhang S (2021). Novel strategy for efficient water splitting through pyro-electric and pyro-photo-electric catalysis of BaTiO_3_ by using thermal resource and solar energy. Appl. Catal. B- Environ..

[5] Xiao L (2021). Pyroelectric nanoplates for reduction of CO_2_ to methanol driven by temperature-variation. Nat. Commun..

[6] Benke A (2015). Pyroelectrically driven •OH generation by barium titanate and palladium nanoparticles. J. Phys. Chem. C..

[7] Gutmann E (2012). Pyroelectrocatalytic disinfection using the pyroelectric effect of nano- and microcrystalline LiNbO_3_ and LiTaO_3_ particles. J. Phys. Chem. C..

[8] Wu J (2016). Strong pyro-catalysis of pyroelectric BiFeO_3_ nanoparticles under a room-temperature cold–hot alternation. Nanoscale.

[9] Raufeisen S, Stelter M, Braeutigam P (2020). Pyrocatalysis—The DCF assay as a pH-robust tool to determine the oxidation capability of thermally excited pyroelectric powders. PLoS ONE.

[10] You H (2018). Piezoelectrically/pyroelectrically-driven vibration/cold-hot energy harvesting for mechano-/pyro-bi-catalytic dye decomposition of NaNbO_3_ nanofibers. Nano Energy.

[11] Baffou G, Cichos F, Quidant R (2020). Applications and challenges of thermoplasmonics. Nat. Mater..

[12] Overgaard J, Hoffmann AA, Kristensen TN (2011). Assessing population and environmental effects on thermal resistance in Drosophila melanogaster using ecologically relevant assays. J. Therm. Biol..

[13] Terblanche JS, Deere JA, Clusella-Trullas S, Janion C, Chown SL (2007). Critical thermal limits depend on methodological context. Proc. R. Soc. B—Biol. Sci..

[14] MacMillan HA, Sinclair BJ (2011). Mechanisms underlying insect chill-coma. J. Insect Physiol..

[15] Vinodgopal K, Stafford U, Gray KA, Kamat PV (1994). Electrochemically assisted photocatalysis. 2. The role of oxygen and reaction intermediates in the degradation of 4-chlorophenol on immobilized TiO_2_ particulate films. J. Phys. Chem..

[16] Baffou G, Bordacchini I, Baldi A, Quidant R (2020). Simple experimental procedures to distinguish photothermal from hot-carrier processes in plasmonics. Light Sci. Appl..

[17] Jauffred L, Samadi A, Klingberg H, Bendix PM, Oddershede LB (2019). Plasmonic heating of nanostructures. Chem. Rev..

[18] Li F (2022). Plasmonic local heating induced strain modulation for enhanced efficiency and stability of perovskite solar cells. Adv. Energy Mater..

[19] Cao L, Barsic DN, Guichard AR, Brongersma ML (2007). Plasmon-assisted local temperature control to pattern individual semiconductor nanowires and carbon nanotubes. Nano Lett..

[20] Liow CH, Lu X, Zeng K, Li S, Ho GW (2019). Optically Governed Dynamic Surface Charge Redistribution of Hybrid Plasmo-Pyroelectric Nanosystems. Small.

[21] Ji Y, Zhang K, Yang Y (2018). A one-structure-based multieffects coupled nanogenerator for simultaneously scavenging thermal, solar, and mechanical energies. Adv. Sci..

[22] Shan D, Pan K, Liu Y, Li J (2020). High fidelity direct measurement of local electrocaloric effect by scanning thermal microscopy. Nano Energy.

[23] You H (2018). Highly efficient pyrocatalysis of pyroelectric NaNbO_3_ shape-controllable nanoparticles for room-temperature dye decomposition. Chemosphere.

[24] Lee D (2009). Mixed Bloch-Néel-Ising character of 180^o^ ferroelectric domain walls. Phys. Rev. B.

[25] Nepochatenko VA, Nepochatenko AV (2020). Comparative analysis of temperature dependencies of the elastic compliance and dielectric permittivity tensor components in BaTiO_3_ single crystal. Ferroelectrics.

[26] Huang H, Sun CQ, Tianshu Z, Hing P (2001). Grain-size effect on ferroelectric Pb(Zr_1-x_Ti_x_)O_3_ solid solutions induced by surface bond contraction. Phys. Rev. B.

[27] Barmina EV, Simakin AV, Shafeev GA (2016). Hydrogen emission under laser exposure of colloidal solutions of nanoparticles. Chem. Phys. Lett..

[28] Chen J (2022). Collective Plasmon Coupling in Gold Nanoparticle Clusters for Highly Efficient Photothermal Therapy. ACS Nano.

[29] Fang Z (2013). Evolution of light-induced vapor generation at a liquid-immersed metallic nanoparticle. Nano Lett..

[30] Govorov AO (2006). Gold nanoparticle ensembles as heaters and actuators: melting and collective plasmon resonances. Nanoscale Res. Lett..

[31] Zhang Y, Xie M, Adamaki V, Khanbareh H, Bowen CR (2017). Control of electro-chemical processes using energy harvesting materials and devices. Chem. Soc. Rev..

[32] Schlechtweg J, Raufeisen S, Stelter M, Braeutigam P (2019). A novel model for pyro-electro-catalytic hydrogen production in pure water. Phys. Chem. Chem. Phys..

[33] de Vivanco MU (2020). Pyroelectrically-driven chemical reactions described by a novel thermodynamic cycle. Phys. Chem. Chem. Phys..

[34] Matés JM, Sánchez-Jiménez FM (2000). Role of reactive oxygen species in apoptosis: implications for cancer therapy. Int. J. Biochem. Cell Biol..

[35] Renschler MF (2004). The emerging role of reactive oxygen species in cancer therapy. Eur. J. Cancer.

[36] Zou Z, Chang H, Li H, Wang S (2017). Induction of reactive oxygen species: an emerging approach for cancer therapy. Apoptosis.

[37] Fu LH (2021). Nanocatalytic theranostics with glutathione depletion and enhanced reactive oxygen species generation for efficient cancer therapy. Adv. Mater..

[38] Xia Y (2017). Pyroelectrically induced pyro-electro-chemical catalytic activity of BaTiO_3_ nanofibers under room-temperature cold–hot cycle excitations. Metals.

