# Supplementary material for: Accelerated pyro-catalytic hydrogen production enabled by plasmonic local heating of Au on pyroelectric BaTiO3 nanoparticles
Source: Nat Commun. 2022 Oct 17;13:6144. doi: 10.1038/s41467-022-33818-4 (PMC9576696; doi:10.1038/s41467-022-33818-4)
Supplement: Supplementary file 1 — Supplementary Information [file 41467_2022_33818_MOESM1_ESM.pdf]

Supporting Information

Accelerated Pyro-Catalytic Hydrogen Production Enabled by Plasmonic Local Heating of Au on Pyroelectric $BaTiO_3$ Nanoparticles

Huilin You[1,6], Siqi Li[1,2,3,6], Yulong Fan[2], Xuyun Guo[1], Zezhou Lin[1], Ran Ding[1], Xin Cheng[4], Hao Zhang[5], Tsz Woon Benedict Lo[1,5], Jianhua Hao[1], Ye Zhu[1], Hwa-Yaw Tam[4], Dangyuan Lei[2,*], Chi-Hang Lam[1], and Haitao Huang[1,*]

1 Department of Applied Physics and Research Institute for Smart Energy, The Hong Kong Polytechnic University, Hong Kong SAR, China.
2 Department of Materials Science and Engineering, The Hong Kong Institute of Clean Energy, The City University of Hong Kong, Hong Kong SAR, China.
3 Information Materials and Intelligent Sensing Laboratory of Anhui Province, Key Laboratory of Opto-Electronic Information Acquisition and Manipulation of Ministry of Education, School of Physics and Materials Science, Anhui University, Hefei, 230601, Anhui, P.R. China.
4 Department of Electrical Engineering, The Hong Kong Polytechnic University, Hong Kong SAR, China.
5 Department of Applied Biology and Chemical Technology, The Hong Kong Polytechnic University, Hong Kong SAR, China.
6 These authors contributed equally: Huilin You and Siqi Li.
*E-Mail: dangylei@cityu.edu.hk (D. Lei) and aphhuang@polyu.edu.hk (H. Huang).

Results and Discussion

The *d*-spacing ($d_{(hkl)}$) of material can be estimated by Bragg's Laws,

$$2d_{(hkl)}\sin\theta_{(hkl)}=n\lambda \tag{1}$$

where n, (hkl), θ and λ are diffraction order (n=1 is adopted), crystallographic orientation (miller indices), Bragg angle (in radius) and wavelength of X-ray, respectively. For tetragonal unit cell, the relation between *d*-spacing and lattice parameters is[1]:

$$\frac{1}{d^2{}_{hkl}}=\frac{h^2+k^2}{a^2}+\frac{l^2}{c^2} \tag{2}$$

By using XRD data and Equations (1) and (2), the calculated lattice parameters are shown in Table S1.

Table S1 Calculated lattice parameters for $BaTiO_3$.

Peak position (2θ)	*d*-spacing	Miller indices	Calculated lattice parameter
45.01	2.012	(002)	c=4.025
45.13	2.007	(200)	a=4.014

Thus, the tetragonality (defined as the ratio of lattice parameters, c/a) of the material can be obtained by c/a=1.003

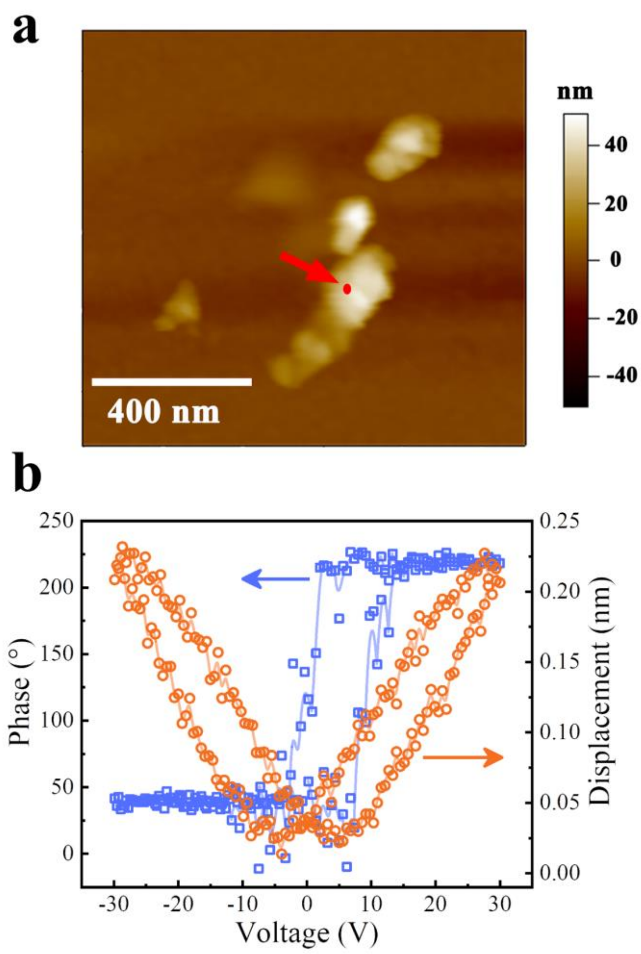

Fig. S1 | PFM characterizations of $BaTiO_3$. a, Topographic image. **b**, Hysteresis loop measurement. Source data are provided as a Source Data file.

The ferroelectric property of $BaTiO_3$ was characterized by PFM, using dual alternating current resonance tracking modes. In PFM characterization, the coral-like shaped $BaTiO_3$ was shattered into separated particles by using ultrasonic vibration, as shown in Fig. S1a and the testing point was marked with a red dot. A butterfly-like hysteresis loop and phase switching can be observed in Fig. S1b. The phase angle

changes about 180° under the reversal of 30 V DC bias field, revealing the ferroelectricity of the $BaTiO_3$.

The energy bandgap (E_g) of $BaTiO_3$ can be estimated by the Tauc relationship and Kubelka-Munk equations[2, 3]:

$$F(R)h\nu=A(h\nu-E_g)^n \tag{3}$$

$$F(R)=\frac{(1-R)^2}{2R} \tag{4}$$

where $F(R)$ is the so-called remission or Kubelka-Munk function, R is reflectance, h is the Planck's constant, ν is the photon frequency, A is a constant, E_g is the energy bandgap, and n is selected as 2 since $BaTiO_3$ is an indirect bandgap material[4,5,6].

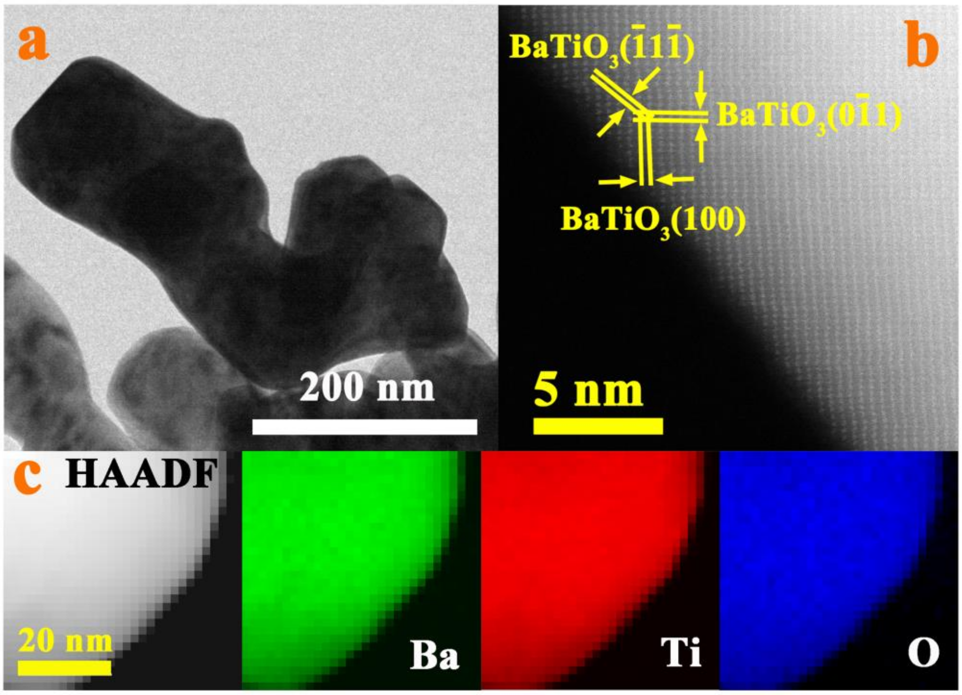

Fig. S2 | Morphology characterizations of three-dimensional hierarchically structured coral-like $BaTiO_3$ NPs. a, TEM image. **b**, High-resolution HAADF-STEM image. **c**, HAADF-STEM image and corresponding elemental mapping of Ba, Ti, and O elements.

The TEM image of the $BaTiO_3$ NP is shown in Fig. S2a. The HAADF-STEM image (Fig. S2b) of the NP shows 3 characteristic lattice spacings, which agree well with the d-spacings of the (100), $(0\bar{1}1)$ and $(\bar{1}1\bar{1})$ planes of $BaTiO_3$. The HAADF-

STEM image and the associated elemental mappings of Ba, Ti and O (Fig. S2c) show uniform distributions of these elements.

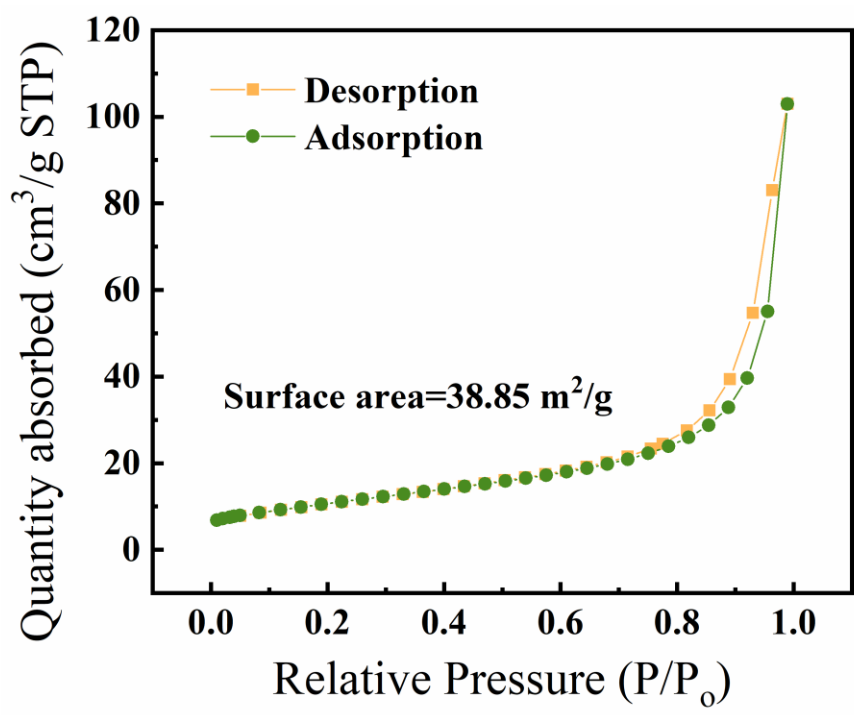

Fig. S3 | Surface area measurement. Surface area characterization of $BaTiO_3$ NPs. Source data are provided as a Source Data file.

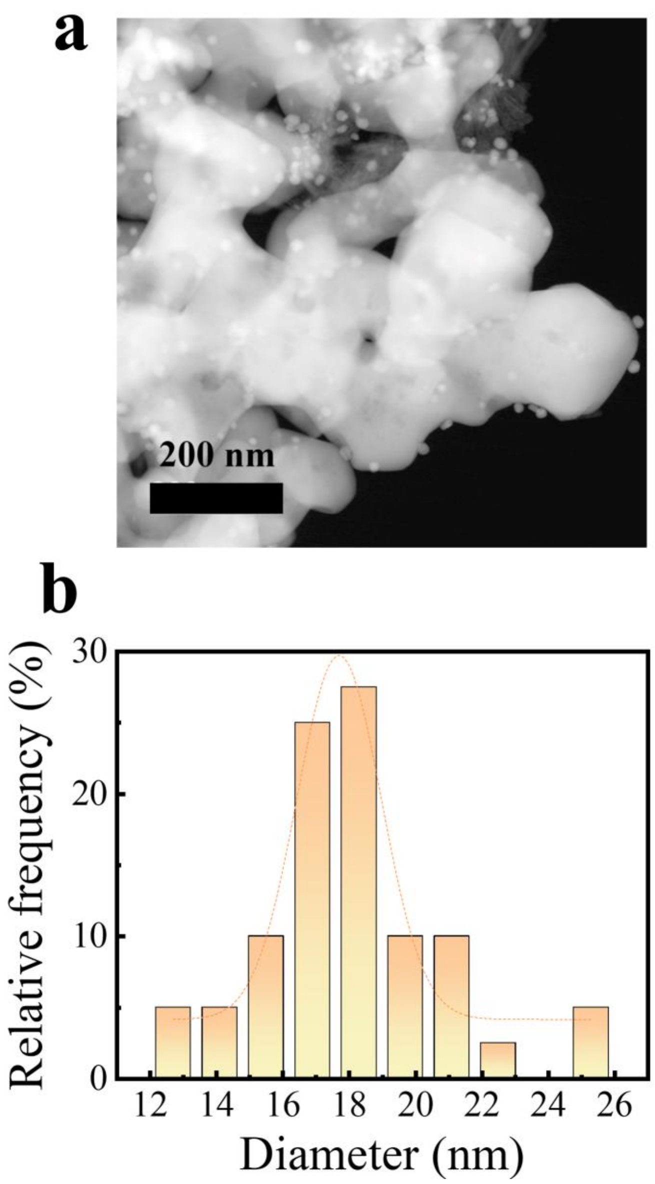

Fig. S4 | Morphology characterization. a, TEM image and **b**, Size distribution of the Au nanospheres. Source data are provided as a Source Data file.

As shown in Fig. S4, the average particle size of the Au nanospheres is found to be abound 18 nm.

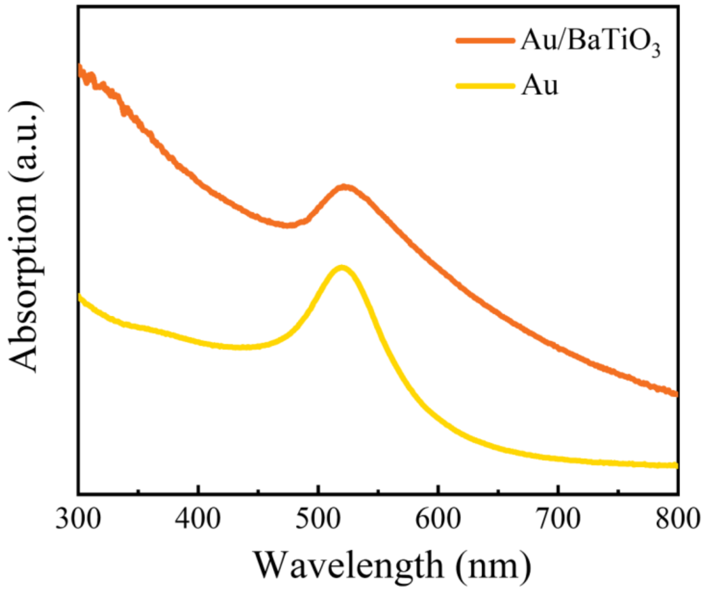

Fig. S5 | Optical absorption. UV-visible absorption spectra of Au nanospheres and Au/$BaTiO_3$ NPs in aqueous solution (radius of Au is 9 nm). Source data are provided as a Source Data file.

Fig. S5 shows UV-visible absorption spectra of Au nanospheres and Au/$BaTiO_3$ NPs in water solution. Au nanospheres showed a narrower surface plasmon resonance band at 532 nm, which is characteristic of individual Au nanospheres, depending on the polydispersity and size of Au nanospheres[7]. The characteristic peak around 532 nm was also observed in Au/$BaTiO_3$ sample, confirming the successful growth of Au nanospheres on $BaTiO_3$ NPs. Due to its large band gap (inset of Fig. 1c), $BaTiO_3$ does not present any noticeable absorption in the visible wavelength range. Comparing with UV-visible absorption spectra of Au nanospheres, the increasing absorption of Au/$BaTiO_3$ NPs below 400 nm may be related to the bandgap absorption of $BaTiO_3$ in the ultraviolet range.

Energy conversion efficiency calculation:

The light-to-chemical energy conversion efficiency is calculated as follows.

The value of the output chemical energy due to pyro-catalysis can be simply calculated as,

$$E_{chem}=2n\cdot V_t\cdot N_A\cdot e \quad (5)$$

where n = mole number of hydrogen produced; V_t = threshold voltage (1.23 V) for water splitting; N_A = Avogadro's number; e = elemental charge.

According to the above equation, the chemical energy output due to pyro-catalysis per second in the present case is:

$$E_{chem}=2n\cdot V_t\cdot N_A\cdot e=2\times9.70\times10^{-11}\times1.23\times6.02\times10^{23}\times1.602\times10^{-19}\ (J\cdot s^{-1})$$

$$=2.30\times10^{-5}\ (J\cdot s^{-1})=0.023\ mW$$

The light-to-chemical energy conversion efficiency can be calculated as,

$$\eta=E_{chem}/E_{input}=0.023\ mW/500\ mW=0.0046\% \qquad (6)$$

This value is quite low since the catalyst used is dispersed in the liquid and the majority of illuminated part is actually water, not the catalyst.

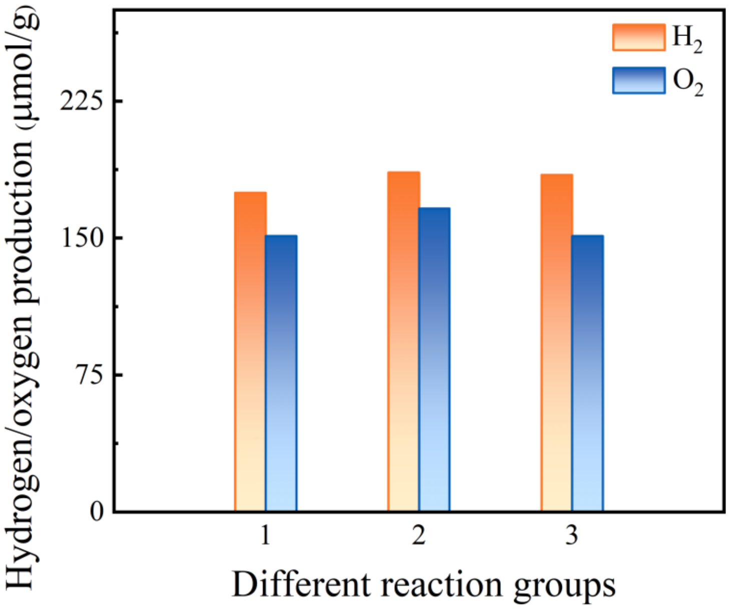

Fig. S6 | Hydrogen production. Full water splitting by Au/$BaTiO_3$ NPs after 90 min irradiation of 532-nm nanosecond pulsed laser. Source data are provided as a Source Data file.

Fig. S6 shows the full water splitting by Au/$BaTiO_3$ NPs under the irradiation of a 532-nm nanosecond laser. The production ratio of H_2 and O_2 gas is less than 2:1, which may result from the air absorbed into the gas-tight syringe during manual injection and testing processes.

Table S2 Hydrogen production via pyro-catalysis

Pyroelectric catalysts	Cold-hot cycle condition	Catalyst dosage (sacrificial agent/concentration)	Catalytic applications	Catalytic efficiency/rate constant	Ref.
BST nanoparticles	298K-323K, 10 min per thermal cycle	10 mg catalyst/10 mL H_2O (20 vol% methanol)	H_2 production	47 $\mu mol \cdot g^{-1}$ (36 thermal cycles)	8
2D few layers BP	288K-338K, 10 min per thermal cycle	1 mg catalyst/10 mL H_2O (20 vol% methanol)	H_2 production	540 $\mu mol \cdot g^{-1}$ (24 thermal cycles)	9
$BaTiO_3$ single crystals	303-343K, 2 min per thermal cycle	3.1 g catalyst (without sacrificial agent)	H_2 production	300 vol.-ppb	10
PZT sheet	316K-319K	4.85 g, thickness of 170 μm, surface area of 49 cm^2 (without sacrificial agent)	H_2 production	0.654 $\mu mol \cdot h^{-1}$	11
SiC	300 K-333 K, 20 min per thermal cycle	50 mg catalyst/20 mL H_2O (10 vol% methanol)	H_2 production	32.84 $\mu mol \cdot g^{-1}$	12
$Au/BaTiO_3$ nanoparticles	500 mW nanosecond laser/10 Hz	2.62 mg catalyst/12 mL H_2O (without sacrificial agent)	H_2 production	133.1±4.3716 $\mu mol \cdot g^{-1}h^{-1}$	This work

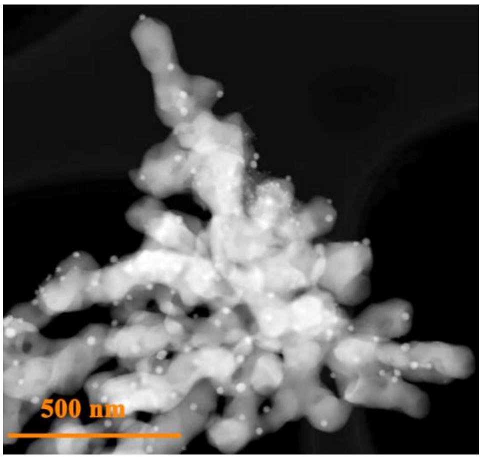

Fig. S7 | TEM image. Morphology of $Au/BaTiO_3$ NPs after pyro-catalysis.

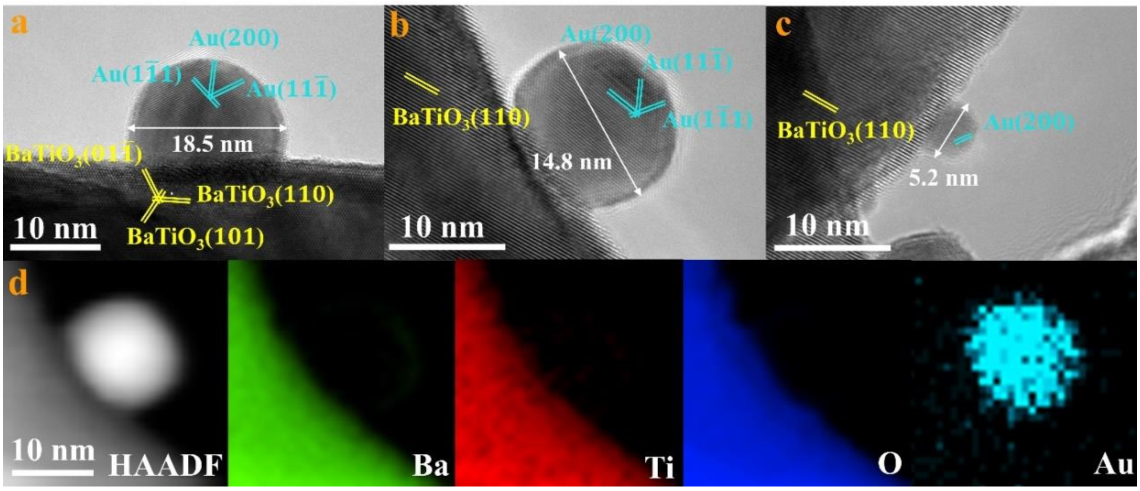

Fig. S8 | Morphology characterizations of $Au/BaTiO_3$ NPs after pyro-catalysis. **a, b and c,** HRTEM image of $Au/BaTiO_3$ NPs after pyro-catalysis with different radii of decorated Au NPs. **d,** HAADF-STEM image and corresponding elemental mapping of Ba, Ti, O, and Au elements of **b**.

As shown in the Fig.S7 and S8, after pyro-catalysis, there is nearly no damage on $Au/BaTiO_3$ NPs.

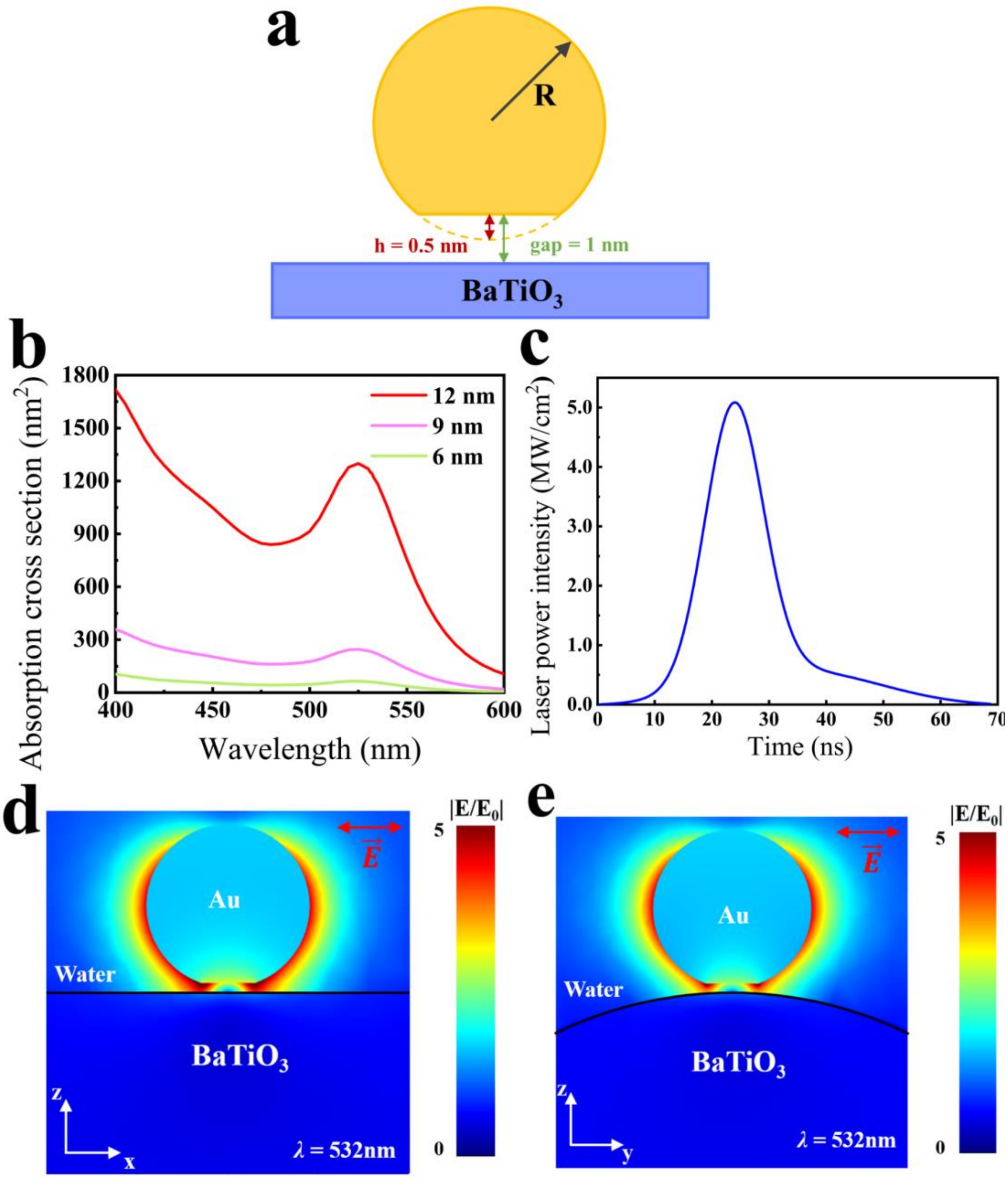

Fig. S9 | Optical simulation. **a**, Structural model for optical simulation: a faceted Au nanosphere (h=0.5 nm) suspending over an $BaTiO_3$ nanowaire (water gap=1nm). **b**, Absorption cross section of an Au nanosphere (in a radius of 6, 9 and 12 nm) suspended over $BaTiO_3$. **c**, Power density of one laser pulse. **d,** Electric field distribution of an Au nanosphere (R= 9 nm as an example) under optical excitation at λ=532 nm with the optical polarization along the radial direction of $BaTiO_3$. **e**, Electric field distribution of an Au nanosphere (R= 9 nm as an example) under optical excitation at λ=532 nm with optical polarization along the axial direction of $BaTiO_3$. Source data are provided as a Source Data file.

A simplified structural model is depicted in Fig. S9a, where *R* denotes the radius and *h* is the height of the sphere cap of Au nanosphere. The refractive index of water is set to be 1.33 and the optical properties of Au and $BaTiO_3$ are taken from Johnson Christy's and Wemple's experimental results, respectively[13,14]. The sphere cap height (h= 0.5 nm) is chosen to reasonably comply with the morphology of the in situ grown Au nanosphere on $BaTiO_3$ NPs. Due to the existence of ligand surrounding an Au nanosphere, the gap between $BaTiO_3$ and the facet of Au nanosphere is chosen to be 1 nm. The absorption cross section peak of the Au nanosphere with different radii are located at 532 nm, as shown in Fig. S9b. The power density used to deduce the heating power of the Au NP during one laser pulse (around 68 ns in duration) is shown in Fig. S9c. The plasmonic modes at excitation wavelength of 532 nm with optical polarizations along radial and axial directions of $BaTiO_3$ are shown in Fig. S9d and S9e, respectively, and are very akin to a dipolar electric mode. To investigate the plasmonic local heating effect during the laser irradiation, the thermal properties of Au nanosphere, $BaTiO_3$ and water are set as follows: the heat capacities of gold and $BaTiO_3$ are 129 and 527 $J \cdot kg^{-1} \cdot K^{-1}$, respectively. The thermal conductivities of gold and $BaTiO_3$ are 317 and 6 $W \cdot m^{-1} \cdot K^{-1}$, respectively. The densities of gold and $BaTiO_3$ are 19300 and 6060 $kg \cdot m^{-3}$, respectively[15]. All the temperature dependent thermal properties of water are taken from the built-in material library of COMSOL Multiphysics 5.5. Using the parameters above, the corresponding temperature distribution of Au/$BaTiO_3$ NP can be obtained.

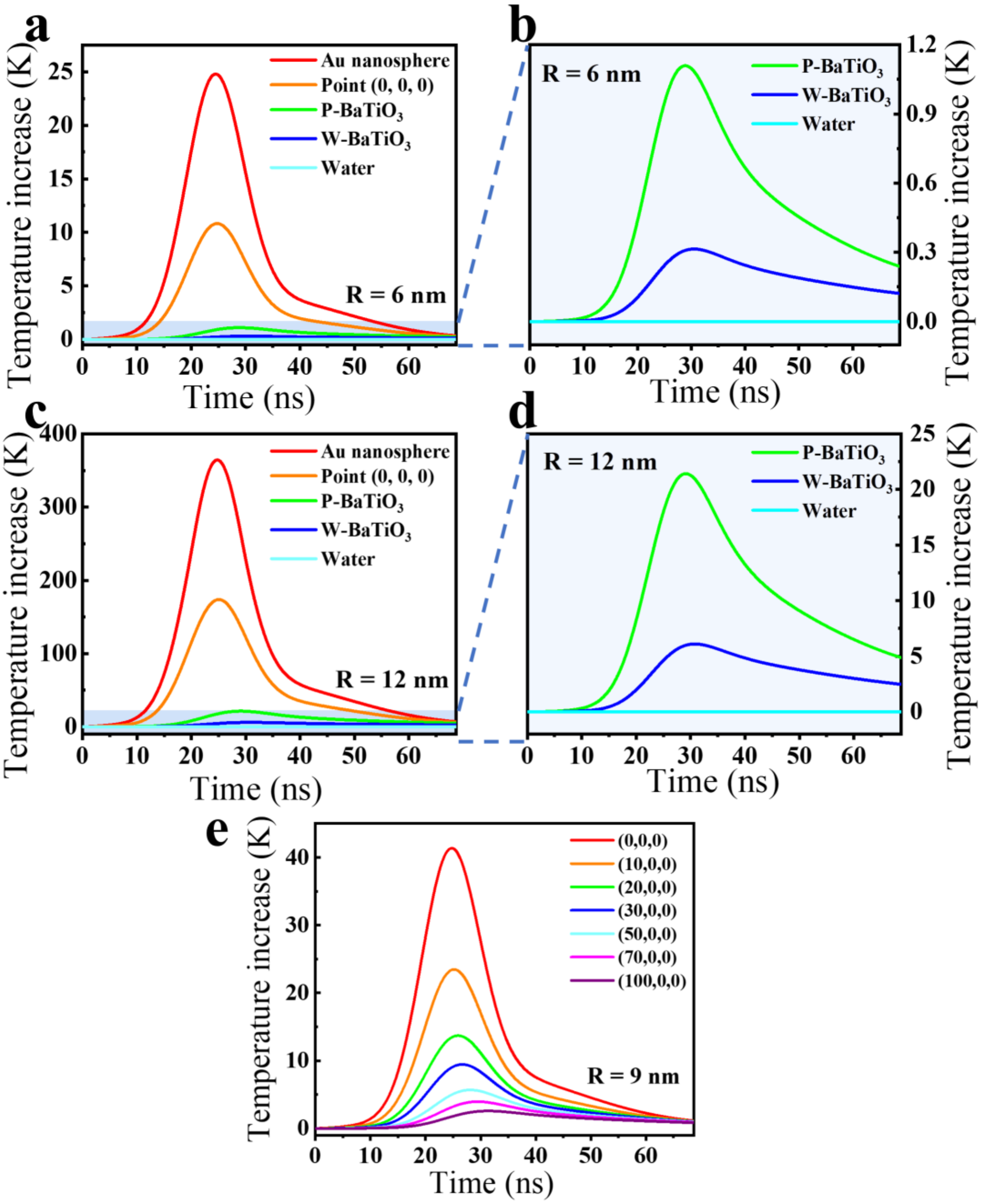

Fig. S10 | Simulated temperature evolution. Time evolution of the temperature of Au nanosphere, point (0, 0, 0), P-$BaTiO_3$, W-$BaTiO_3$ and surrounding water with different radii of decorated Au NPs: **a**, 6 nm and **c**, 12 nm. Zoom-in time evolution of the temperature of P-$BaTiO_3$, W-$BaTiO_3$, and surrounding water with different radii of decorated Au NPs: **b**, 6 nm and **d**, 12 nm. **e**, Time evolution of the temperature increase along axial direction of a $BaTiO_3$ NP decorated with a 9-nm Au nanosphere. Source data are provided as a Source Data file.

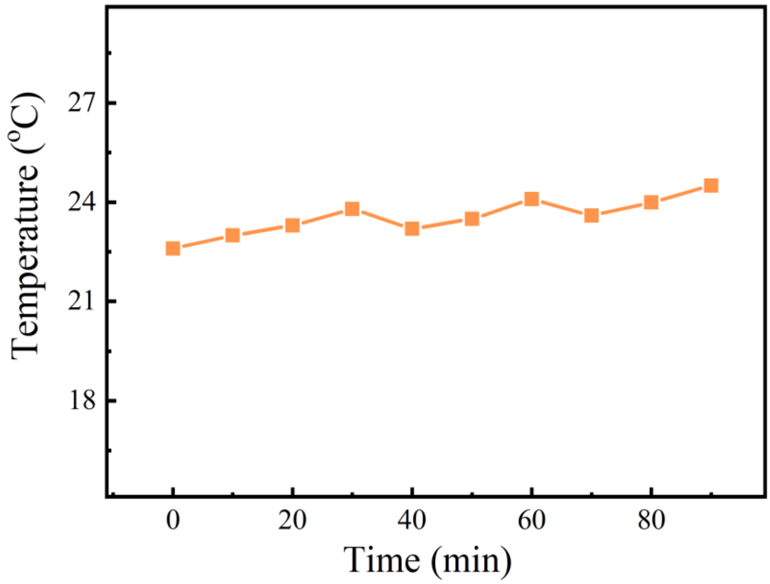

Fig. S11 | Water temperature. Temperature variation of water under prolonged irradiation of nanosecond laser during the plasmon induced pyro-catalysis. Source data are provided as a Source Data file.

As shown in Fig. S11, temperature change of water during plasmon induced pyro-catalysis is negligible. The overall temperature of water was monitored by a thermometer.

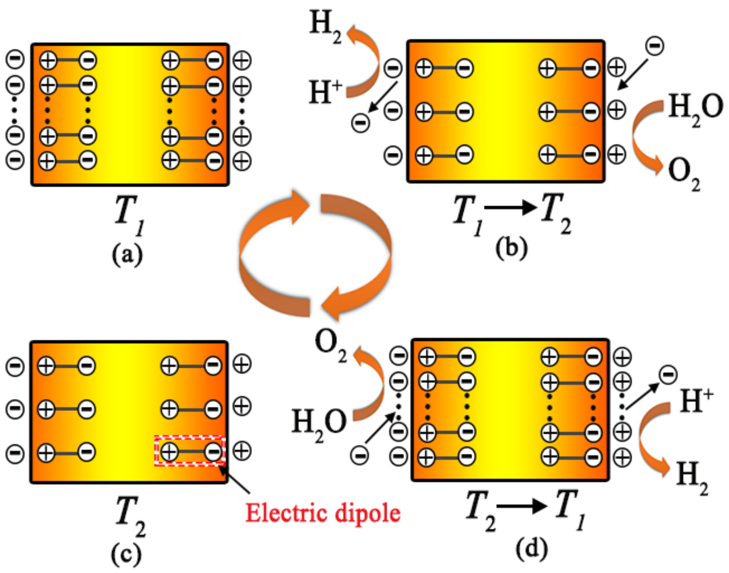

Fig. S12 | Mechanism of pyro-catalysis. a, equilibrium polarization state at temperature T_1. **b,** the temperature is raised from T_1 to T_2. **c,** equilibrium polarization state at temperature T_2. **d,** the temperature is decreased from T_2 to T_1.

The source of charges mainly comes from two origins: (1) free electrons in the conduction band, and (2) screen charges on the surface[16]. The free electrons may come from defects (such as oxygen vacancies) to keep neutrality of the material's internal charges. In the present case, the hot electrons injected from the Au nanoparticles to $BaTiO_3$ particles is another source of free electrons. Those free electrons will drift under the pyroelectrically induced internal field and migrate to the surface to participate in the catalytic reaction. The surface screen charges come from charged species in the liquid to compensate the polarization-induced surface bound changes. Under thermal fluctuation, the spontaneous polarization may vary and the redundant screen charges may participate in the catalytic reaction.

Fig.S12 schematically illustrates the pyro-catalysis mechanism. Initially, both free electrons and screen charges will accumulate at the surface of nanoparticles to compensate the polarization-induced surface bound changes (Fig.S12a). When the temperature is raised from T_1 to T_2, the spontaneous polarization is reduced. Hence the positively polarized surface will release electrons to have the hydrogen evolution reaction (HER) and the negatively polarized surface will accept electrons from water molecules to have the oxygen evolution reaction (OER) (Fig.S12b). Fig.S12c depicts the equilibrium state at the higher temperature of T_2. When the temperature is decreased from T_2 to T_1, the spontaneous polarization is increased. Hence the whole process is

reversed, that is, the positively polarized surface will accept electrons to have OER and the negatively polarized surface will release electrons to have HER (Fig.S12d).

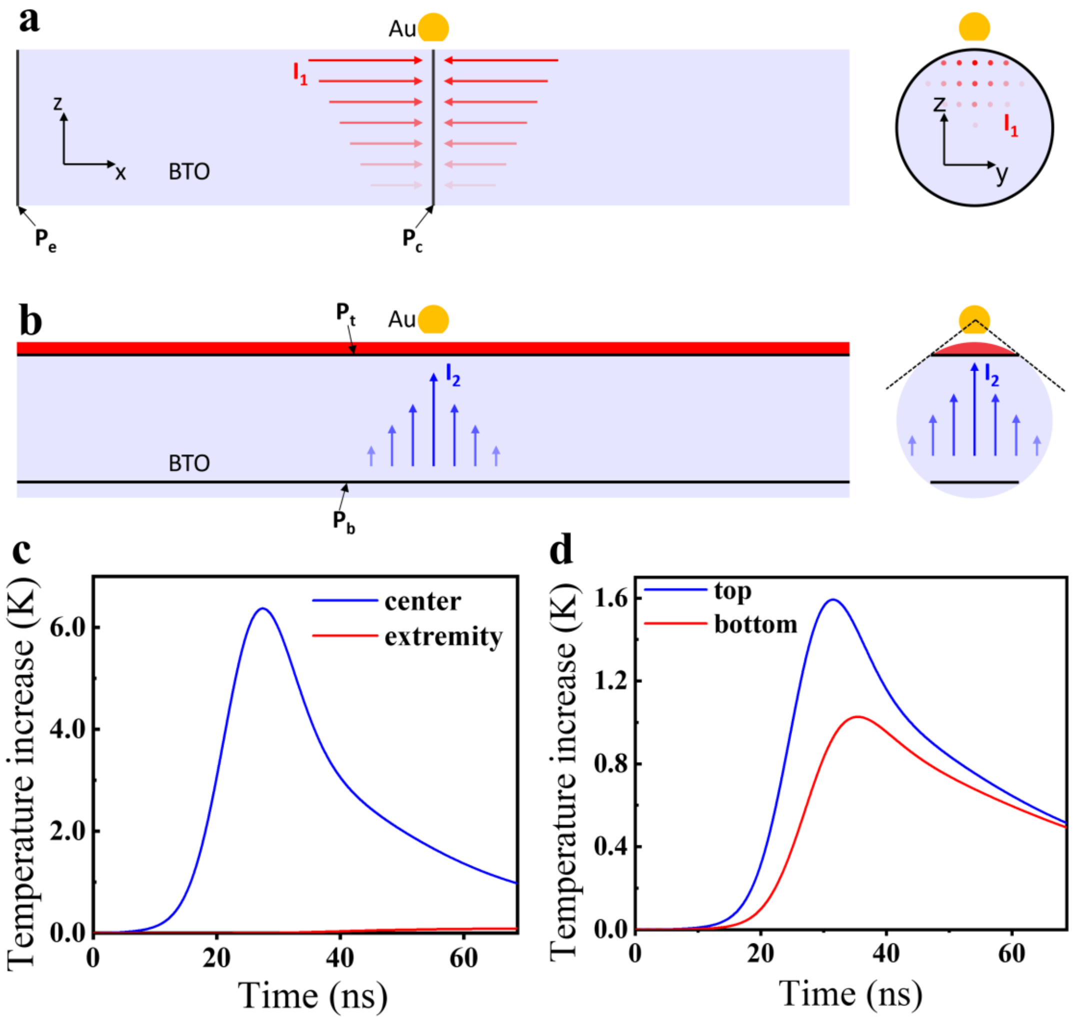

Fig. S13 | Optical Simulation. Structural model for simulation: **a**, One faceted Au nanosphere (h = 0.5 nm) suspending over a cylindrical $BaTiO_3$ particle (water gap = 1 nm). Left panel: cut planes used to calculate the temperature difference in axial direction (their positions are represented by black lines and marked as $\mathbf{P_c}$ and $\mathbf{P_e}$). Right panel: side-view of $\mathbf{P_c}$ and $\mathbf{P_e}$ cut planes. **b,** The same schematic as that shown in **a**, but with two different cut planes, i.e., $\mathbf{P_t}$ and $\mathbf{P_b}$, used to calculate the temperature difference in *z*-direction. **c**, Surface-averaged temperature of $\mathbf{P_c}$ (blue curve, marked as "center") and $\mathbf{P_e}$ (red curve, marked as "extremity"). **d**, Surface-averaged temperature of $\mathbf{P_t}$ (blue curve, marked as "top") and $\mathbf{P_b}$ (red curve, marked as "bottom"). Source data are provided as a Source Data file.

To calculate the thermoelectrically induced charges, we set two circular cut planes,

marked as $\mathbf{P_c}$ and $\mathbf{P_e}$ in Fig. S13a. The temperature difference between the two planes ($\mathbf{P_c}$ and $\mathbf{P_e}$) will induce current flows due to thermoelectric effect in the axial direction of the cylindrical particle, as marked by red arrows. The length of red arrow schematically shows the magnitude of current density (not drawn to scale, Fig. S13a left panel). Similarly, we also consider the current flows in a direction perpendicular to the axial direction, as marked by blue arrows in Fig. S13b left panel. Two cut planes parallel to the *xy*-plane are created within the cylindrical $BaTiO_3$ particle. By drawing tangent lines from the center of the Au nanosphere to the $BaTiO_3$ surface (Fig. S13b, right panel), the plane $\mathbf{P_t}$ is formed by all the points of tangency. Simple calculation shows that $\mathbf{P_t}$ is 7.98 nm from the top of the cylindrical $BaTiO_3$ particle. For symmetry consideration, the other cut plane $\mathbf{P_b}$ is parallel to $\mathbf{P_t}$ but at an equal distance of 7.98 nm above the bottom of $BaTiO_3$ particle, serving as a reference plane for calculating the temperature difference. These two planes are reasonably chosen to evaluate the thermoelectric effect in *z*-direction since the $BaTiO_3$ surface above $\mathbf{P_t}$ (highlighted by red colored zone in Fig. S13b) is regarded as being directly heated by the Au nanosphere and thus be treated as the heat source in the thermal process. The surface-averaged temperature in the $\mathbf{P_c}$ and $\mathbf{P_e}$ cut planes are given in Fig. S13c. And the surface-averaged temperatures in the $\mathbf{P_t}$ and $\mathbf{P_b}$ cut planes are given in Fig. S13d. It can be seen that, the temperature difference in axial direction is much higher as $\mathbf{P_c}$ and $\mathbf{P_e}$ cut planes are far apart. On the contrary, the surface-averaged temperature difference in $\mathbf{P_t}$ and $\mathbf{P_b}$ cut planes is much smaller due to their smaller separation distance (84.04 nm).

The quantity of electrons induced by thermoelectric effect can be derived as follows[17]:

$$Q = \int Idt = \int \sigma(T)EdAdt = \int \sigma(T)\frac{V}{l}dAdt = \int S\frac{\sigma(T)\Delta T}{l}dAdt \qquad (7)$$

Where I, σ, E, V, l, A, t, S, and ΔT are the current, the electrical conductivity[18], electric field, thermoelectric voltage, the distance between two cut planes, surface area, time, Seebeck coefficient and the temperature difference between two end planes, respectively.

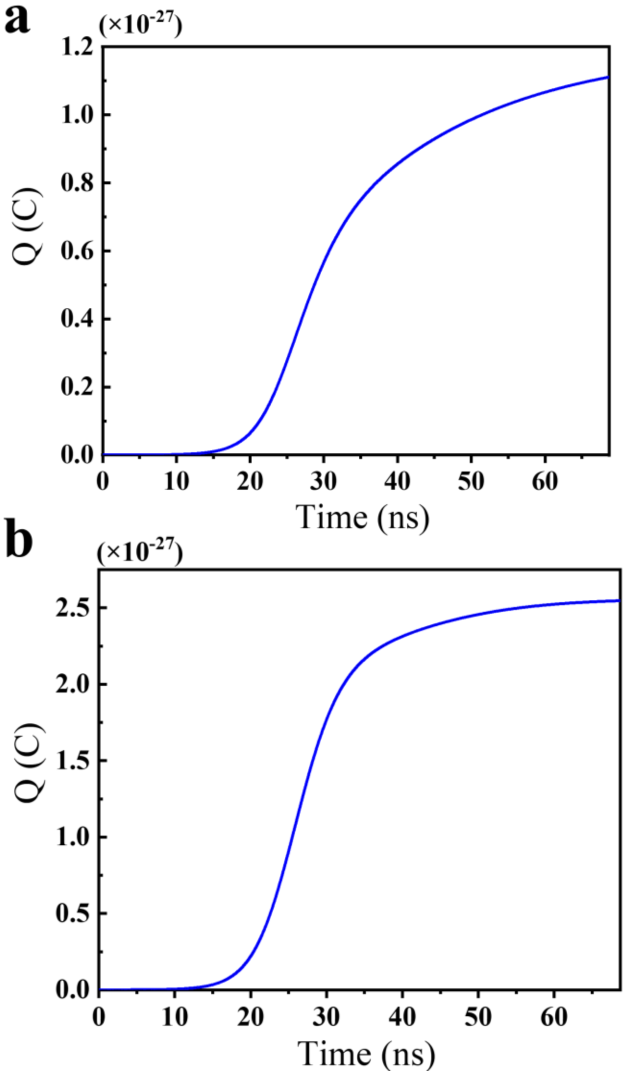

Fig. S14 | Simulation of thermoelectric effect. Quantity of electric charges induced by thermoelectric effect in **a**, axial direction; and **b**, z-direction. Source data are provided as a Source Data file.

It should be noted that although the surface-averaged temperature difference in $\mathbf{P_t}$-$\mathbf{P_b}$ cut planes is much smaller than that in $\mathbf{P_c}$-$\mathbf{P_e}$ cut planes (Fig.S13c and Fig.S13d), the larger surface area of $\mathbf{P_t}$-$\mathbf{P_b}$ (542000 nm^2 *vs*. 7584 nm^2) and their smaller separation distance (84.04 nm *vs*. 500 nm) finally result in a larger value in electrons induced by thermoelectricity (Fig.S14).

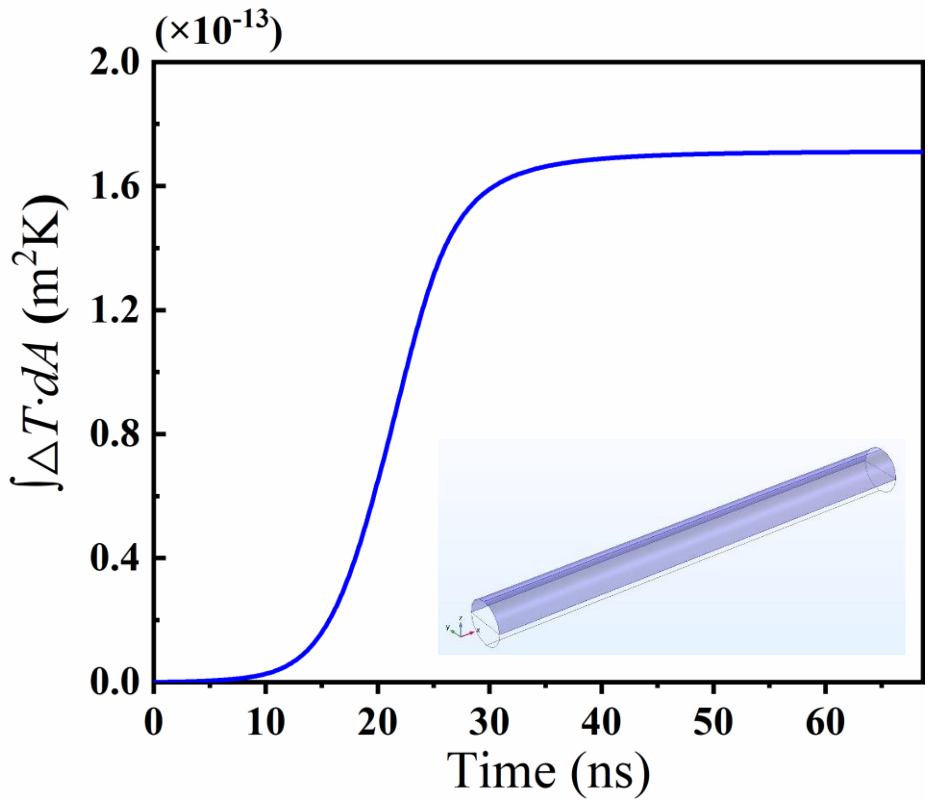

Fig. S15 | Simulated temperature change. Area integrated temperature change ($\int \Delta T(\hat{\mathbf{n}} \cdot \hat{\mathbf{P}}) dA$) over the upper half surface of the cylindrical $BaTiO_3$ particle after one laser pulse irradiation on a 9-nm Au NP. Inset shows the integrated area (shaded part) of the particle. Source data are provided as a Source Data file.

The total pyroelectric charges over the surface of $BaTiO_3$ NPs within the heating process during one pulse irradiation on a 9-nm Au NP can be calculated by:

$$Q = p \cdot \int \Delta T(\hat{\mathbf{n}} \cdot \hat{\mathbf{P}}) dA \quad (8)$$

where p is the pyroelectric coefficient with a value between 20 and 30 $nC \cdot cm^{-2} \cdot K^{-1}$, ΔT is the temperature rise in heating (or drop during cooling), $\hat{\mathbf{n}}$ is the unit vector along the surface normal direction, and $\hat{\mathbf{P}}$ is the unit vector along spontaneous polarization direction.

Without losing generality, we compare the cases for polarizations along the axial direction and z-direction. The integral of $\Delta T(\hat{\mathbf{n}} \cdot \hat{\mathbf{P}}) dA$ over the upper surface (corresponding polarization along z-axis) of the cylindrical particle can be up to 1.71×10^{-13} m^2K (Fig. S15), which is much larger than the integral over the $\mathbf{P_c}$ cut plane (corresponding polarization along axial direction) (location of $\mathbf{P_c}$ can be found from Fig. S13a). Moreover, the pyroelectric charges generated on the $\mathbf{P_c}$ cut plane are difficult to

diffuse to the surface for catalytic reaction due to the low conductivity of $BaTiO_3$. Therefore, we consider only the polarization along *z*-axis for the estimation of the upper limit of H_2 production.

Taking the pyroelectric coefficient (*p*) of 30 $nC\cdot cm^{-2}\cdot K^{-1}$ as an example, we can easily calculate that the pyroelectric charges are 5.12×10^{-17} C, which are much larger than those due to thermoelectric effect (Fig. S14). So the thermoelectric effect induced catalysis can be neglected in the present case.

The above results show that the maximum amount of pyroelectric charges produced over the surface of one $BaTiO_3$ NP by one single Au NP during the heating process by one-pulse illumination is about 5.12×10^{-17} C, which is equivalent to 320 electrons. The pyroelectric charges produced over the lower half surface of the cylinder during the cooling process may also contribute to the catalytic H_2 production. They are estimated to be around 60 % of that produced from the upper half surface during the heating process since the average temperature change of the lower half surface is around 60% of that of the upper half surface (Fig. S13d). So the total available pyroelectric charges for pyro-catalysis during one complete heating/cooling cycle is 512 electrons.

In the present case, the repetition rate of the laser is 10 Hz. Hence, the maximum number of electrons produced over the surface of one $BaTiO_3$ NP after one-hour nanosecond laser irradiation on one 9-nm Au NP for pyro-catalysis ($N_{one\ particle}$) can be calculated as,

$$N_{one\ particle}=512\times10\times60\times60=1.84\times10^{7} \quad (9)$$

The total number of $BaTiO_3$ NPs in 1 gram is,

$$N=\frac{m}{\rho\cdot V}=\frac{1g}{6060kg\cdot m^{-3}\times\pi\times50nm\times50nm\times1000nm}=2.09\times10^{13} \quad (10)$$

where ρ is density of $BaTiO_3$ (= 6060 $kg\cdot m^{-3}$).

Thus, after one-hour nanosecond laser irradiation, for 1 g of $BaTiO_3$ NPs, the total number available electrons for pyro-catalysis is $n=N_{one\ particle}\times N=3.85\times10^{20}$. Therefore, the pyro-catalytic hydrogen production rate (R) over the surface of $BaTiO_3$ NPs is

$$R = \frac{n}{2N_A} = \frac{3.85 \times 10^{20}}{2 \times 6.02 \times 10^{23}} = 319.8\ \mu\text{mol} \cdot \text{g}^{-1} \cdot \text{h}^{-1} \qquad (11)$$

where N_A is the Avogadro's number.

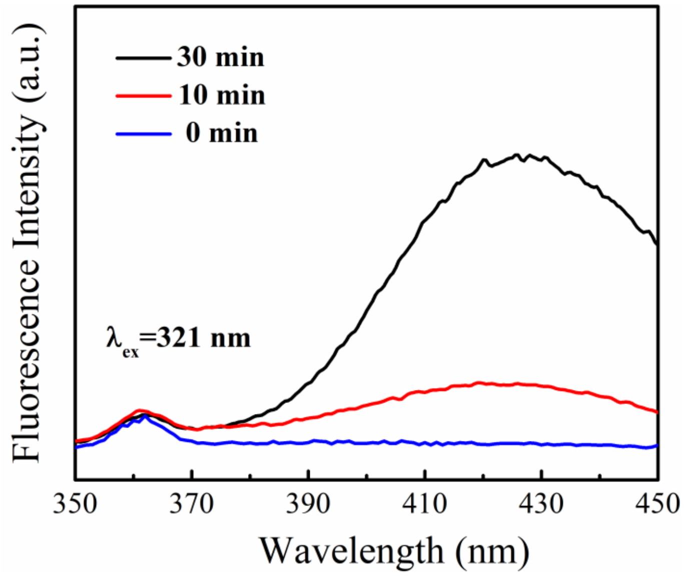

Fig. S16 | Trapping of hydroxyl radical. Fluorescence spectra for •OH trapping after experiencing 0, 10, and 30 min pyro-catalysis reaction time. Source data are provided as a Source Data file.

During the pyro-catalytic reaction, hydroxyl radicals (•OH) can also be generated, which was captured by a photoluminescent •OH trapping agent, terephthalic acid. Terephthalic acid can react with •OH to produce a highly fluorescent product, 2-hydroxyterephthalic acid, which emits a unique fluorescence signal with its peak wavelength at 425 nm[19]. The PL intensity of 2-hydroxyterephtalic acid relies on the amount of •OH generated in water[19]. This sensitive and specific method has been widely used for the •OH detection[20,21,22]. In the current work, around 0.5 mg Au/$BaTiO_3$ NPs were dispersed in 4 mL terephthalic acid aqueous solution (0.5 mM) with a concentration of 2 mM NaOH in a quartz reactor. After experiencing various irradiation time by a 532 nm nanosecond laser, the PL spectra of the generated 2-hydroxyterephthalic acid was measured via a spectrofluorometer under an excitation wavelength of 321 nm. In Fig. 16, with increasing reaction time, the 425 nm fluorescence intensity gradually increases, which indicates the increasing amount of •OH radicals synthesized under pyro-catalysis.

Supplementary References

1. Bae, H. & Lee, K. T. Effect of tetragonal to cubic phase transition on the upconversion luminescence properties of A/B site erbium-doped perovskite $BaTiO_3$. *RSC Adv.*, **9**, 2451-2457 (2019).

2. Ke, J. *et al.*, Facile assembly of $Bi_2O_3/Bi_2S_3/MoS_2$ np heterojunction with layered n-Bi_2O_3 and p-MoS_2 for enhanced photocatalytic water oxidation and pollutant degradation. *Appl. Catal. B- Environ.* **200**, 47-55 (2017).

3. Kresse, G. & Furthmuller, J. Efficiency of ab-initio total energy calculations for metals and semiconductors using a plane-wave basis set. *Comput. Mater. Sci.*, **6**, 15-50 (1996).

4. Upadhyay, S. *et al.*, Enhanced photoelectrochemical response of $BaTiO_3$ with Fe doping: experiments and first-principles analysis. *J. Phys. Chem. C*, **115**, 24373-24380 (2011).

5. Ali, A., Khan, I., Ali, Z., Khan, F. & Ahmad, I. First-principles study of $BiFeO_3$ and $BaTiO_3$ in tetragonal structure. *Int. J. Mod. Phys. B*, **33**, 1950231 (2019).

6. Rizwan, M. *et al.*, Electronic, structural and optical properties of $BaTiO_3$ doped with lanthanum (La): Insight from DFT calculation. *Optik*, **211**,164611 (2020).

7. Cavicchi, R. E., Meier, D. C., Presser, C., Prabhu, V. M. & Guha, S. Single laser pulse effects on suspended-Au-nanoparticle size distributions and morphology. *J. Phys. Chem. C,* **117**, 10866-10875 (2013).

8. Xu, X. *et al.*, Pyro-catalytic hydrogen evolution by $Ba_{0.7}Sr_{0.3}TiO_3$ nanoparticles: harvesting cold–hot alternation energy near room-temperature. *Energy Environ. Sci.*, **11**, 2198-2207 (2018).

9. You, H. *et al.*, Room-temperature pyro-catalytic hydrogen generation of 2D few-layer black phosphorene under cold-hot alternation. *Nat. commun.* **9**, 1-8 (2018).

10. Belitz, R. *et al.*, Waste heat energy harvesting by use of $BaTiO_3$ for pyroelectric hydrogen generation. *Energy Harvesting & Systems* **4**, 107-113 (2017).

11. Zhang, Y. *et al.*, Pyro-electrolytic water splitting for hydrogen generation. *Nano Energy*, **58**, 183-191 (2019).

12. Sun, S., Song, L., Zhang, S., Sun, H. & Wei, J. Pyroelectric hydrogen production performance of silicon carbide. *Ceram. Int.*, **47**, 20486-20493 (2021).

13. P. B. Johnson & R. W. Christy, Optical constants of the noble metals. *Phy. Rev. B,* **6**, 4370 (1972).

14. Wemple, S. H., Didomenico, Jr. M. & Camlibel, I. Dielectric and optical properties of melt-grown $BaTiO_3$. *J. Phys. Chem. Solids*, **29**,1797-1803 (1968).

15. Habenicht, A., Olapinski, M., Burmeister, F., Leiderer, P. & Boneberg, J. Jumping nanodroplets. Science, **309**, 2043-2045 (2005).

16. Wang, K. *et al.*, The mechanism of piezocatalysis: energy band theory or screening charge effect? *Angew. Chem. Int. Ed.* **61**, e202110429 (2022).

17. Mennemanteuil, M. M. *et al.*, Laser-induced thermoelectric effects in electrically biased nanoscale constrictions. *Nanophotonics*, 7, 1917-1927 (2018).

18. Mme GODEFROY G. and M. COUSON B., Conductivity op $BaTiO_3$. pure single crystals and doped (Fe, OH) single crystals. *J. Phys. Colloques,* **33**, C2-120-C2-122

(1972).
19. Ishibashi, K., Fujishima, A., Watanabe, T. & Hashimoto, K. Detection of active oxidative species in TiO_2 photocatalysis using the fluorescence technique. *Electrochem. Commun.* **2**, 207-210 (2000).
20. Palaniswamy, V. K., Ramasamy B., Manoharan, K., Raman, K. & Sundarama, R. Enhanced photocatalytic degradation of tetracycline antibiotic using m-$BiVO_4$ photocatalyst under visible light irradiation. *Chem. Phys. Lett.*, **771**, 138531 (2021).
21. Cheng, X., Liu, H., Chen, Q., Li, J. & Wang, P. Preparation of graphene film decorated TiO_2 nano-tube array photoelectrode and its enhanced visible light photocatalytic mechanism. *Carbon*, **66**, 450-458 (2014).
22. de Mendonça, V. R., Lopes, O. F., Fregonesi, R. P., Giraldi, T. R. & Ribeiro, C. TiO_2-SnO_2 heterostructures applied to dye photodegradation: The relationship between variables of synthesis and photocatalytic performance. *Appl. Surf. Sci.* **298**, 182-191 (2014).
